# Reinsurance–investment game between two *α*-maxmin mean–variance insurers

**DOI:** 10.1371/journal.pone.0326125

**Published:** 2025-06-27

**Authors:** Qian Zhang, Guoyong Zhou, Jing Fu

**Affiliations:** 1 School of Mathematics and Physics, Leshan Normal University, Leshan, Sichuan, China; 2 School of Mathematics and Physics, Chongqing University of Science and Technology, Chongqing, China; Roma Tre University: Universita degli Studi Roma Tre, ITALY

## Abstract

This paper examines a non-zero-sum stochastic differential reinsurance-investment game between two competitive insurers under the α-maximin mean-variance criterion. Both insurers can purchase proportional reinsurance and invest in a financial market consisting of one risk-free asset and one risky asset, and each insurer is concerned with its terminal surplus and relative performance compared to its competitor. The insurers aim to maximize the α-maximin mean-variance utility, which allows them to exhibit different attitudes towards model ambiguity. By solving the extended Hamilton-Jacobi-Bellman (HJB) equations for both insurers, we derive the α-robust equilibrium reinsurance and investment strategies. Finally, several numerical examples are provided to illustrate the impact of some model parameters on the equilibrium strategies.

## 1 Introduction

Reinsurance and investment are the two main issues in actuarial science. In recent years, extensive research has been conducted on these topics with a variety of objectives, which include minimizing the probability of ruin, as explored by [1–3], etc.; maximizing the expected utility of the terminal wealth, as investigated by [4–6], etc.; and applying the mean-variance criterion, as studied by [7–9], etc.

However, most studies assume that insurers can accurately estimate the surplus process and financial risk asset fluctuations. In reality, financial and insurance markets are fraught with uncertainties beyond the scope of insurers’ cognition, making accurate model estimation challenging. Consequently, many scholars have introduced uncertainty into the model, known as the robust control problem. For example, [10] assume that the insurer is ambiguity-averse, its surplus process follows the Cramér-Lundberg model, and derive the robust investment and proportional reinsurance strategy under the mean–variance criterion. [11] assume that an ambiguity-averse insurer (AAI) can invest its wealth in a market index, a risk-free asset, and a pair of mispriced stocks, and derive the robust strategy. For more literature, the readers may refer to [12–14], etc.

The above literature on robust control problems assume that insurers are extremely ambiguity-averse and inclined to consider the strategies in the worst-case scenario. In fact, very few decision makers are extremely ambiguity-averse. The experiments conducted by [15] show that agents’ attitudes towards ambiguity can range from mild ambiguity-aversion to ambiguity-seeking when they believe they have more experience or a better understanding of the actual situation. Considering different attitudes towards the ambiguity, [16,17] propose a more general utility function called α-maxmin expected utility. Then [18] assume that the insurer’s surplus process is correlated with the dynamics of the risky asset, adopt the new mean-variance criterion, namely α-maxmin mean-variance criterion, to the reinsurance-investment problem, and obtain the optimal strategy. [19] generalize [18] by loosing the restriction on the parameter α and the reinsurance form. Considering the delay feature, [20] study the α-robust reinsurance–investment problem under Heston’s stochastic volatility model.

As we known, the financial institutions often concern the performance of their competitors when they make decisions. Therefore, some scholars study the non-zero-sum stochastic differential reinsurance-investment game between two competitive insurers. [21] formulate the non-zero-sum reinsurance-investment game between two insurers and derive the equilibrium strategies using dynamic programming principles. In [22], the surplus processes of two competing insurers are modeled by the Cramér-Lundberg models and diffusion approximated models, respectively. Under the mean-variance criterion, they study the robust non-zero-sum game and obtain the time-consistent reinsurance-investment equilibrium strategies.

To the best of our knowledge, there exists rare literature studying on the robust non-zero-sum stochastic differential game between two competing insurers under the α-maxmin mean-variance criterion. In this paper, we aim to explore how concerns over relative performance and attitudes towards ambiguity affect the equilibrium reinsurance and investment strategies. Specifically, the surplus process of each insurer is described by a diffusion approximation model which has been widely used in the literature, and each insurer can manage its risk by purchasing proportional reinsurance from a reinsurer, and invest in a financial market consisting of one risk-free asset and one risky asset. By applying the principle of dynamic programming, we establish the extended Hamilton-Jacobi-Bellma (HJB) equations, and derive the α-robust time-consistent optimal reinsurance-investment strategies. The main innovations are concluded as follows. First, we extend the α-robust optimal reinsurance–investment problem, previously studied for a single insurer in [18], to a stochastic differential game involving two competitive insurers, and we find that competition makes insurers more risk-seeking. Second, we incorporate the α-maxmin mean–variance criterion into the non-zero-sum reinsurance-investment game, and we get the closed-form strategies and value functions. Third, we present a verification theorem for the α-maxmin-variance non-zero-sum game, which is a valuable addition to the existing literature.

The remainder of this article is structured as follows. Section 2 formulates the non-zero-sum stochastic differential reinsurance-investment game between two competitive insurers under the α-maxmin mean–variance criterion. In Section 3, we derive the equilibrium strategies and value functions by solving the extended HJB equations. Numerical examples are presented in Section 4 to illustrate the impact of key model parameters on the equilibrium strategies. Finally, Section 5 concludes the article.

## 2 Mathematical model

Let $\left(\Omega ,ℱ,\mathbb{P},\{{ℱ}_{t}{\}}_{t\in [0,T]}\right)$ be a complete probability space satisfying the usual conditions of completeness and right continuity, where *T* > 0 represents a fixed finite time horizon. Assume that all stochastic processes below are well-defined and adapted in the space.

### 2.1 Wealth process

In this paper, we suppose the insurance market consists of two competing insurers, who are abbreviated as insurer 1 and insurer 2. Assume that the surplus process of insure *k*, k∈{1,2}, without reinsurance is modeled by the diffusion approximation (DA) model as

$$dR_{k}(t)=\mu_{k}dt+\sigma_{k}dW_{k}(t),\;k\in \{1,2\},$$ (1)

where the parameters $\mu_{k}>0$ and $\sigma_{k}>0$ are the premium return rate and the volatility, respectively. *Wk*(*t*) is a standard Brownian motion, and satisfies $d<W_{1}(t),W_{2}(t)>=\rho dt$, where 0<ρ<1 which captures the correlation of the two insurers’ businesses. Readers who are interested in the derivation process of the DA model can refer to [23]. The DA model (1) is highly effective in the context of large insurance portfolios, as each claim is relatively insignificant when compared to the overall surplus size, and this model has been widely used in the literature, for example, [1,24–26], etc.

Assume that the two insurers can manage their insurance risks through purchasing proportional reinsurance or acquiring new insurance businesses. Denote the risk exposure of insurer *k* at time *t* by $q_{k}(t):[0,T]\to [0,+\infty )$. When $q_{k}(t)\in [0,1]$, it indicates that insurer *k* purchases reinsurance from a reinsurer. In this case, the risk exposure of insurer *k* reduces to $100q_{k}\%$, meanwhile, the reinsurer would indemnify the rest $100(1\;-\;q_{k})\%$ of each claim and charge a reinsurance premium at a rate of $p_{k}^{q_{k}}(t)=(1\;-\;q_{k}(t))\eta_{k}$, where $\eta_{k}>\mu_{k}>0$ is the premium return rate of the reinsurer. When $q_{k}(t)\in (1,+\infty )$, it means that insurer *k* acts as a reinsurer and obtains new business from other insurers (refer to [27]). Under the reinsurance strategy *q*_*k*_(*t*), the surplus process of insurer *k* becomes


$$dU_{k}(t)=[\lambda_{k}+\eta_{k}q_{k}(t)]dt+\sigma_{k}q_{k}(t)dW_{k}(t),$$

where $\lambda_{k}=\mu_{k}-\eta_{k}$.

We further assume that the two competing insurers can invest in a financial market consists of one risk-free asset and one risk stock. The price dynamics *S*0(*t*) of the risk-free asset is described by


$$dS_{0}(t)=rS_{0}(t)dt,$$

where *r*_0_>0 is the risk-free interest rate. The price of the risk stock, denoted by *S*(*t*), evolves according to the geometric Brownian motion (GBM), which is employed in the Black–Scholes model for stock price modeling. GMB is a continuous-time stochastic process in which the logarithm of the randomly varying quantity follows a Brownian motion with drift. In real stock prices, volatility is a dynamic variable that changes over time. However, GBM simplifies the situation by assuming a constant volatility. Additionally, stock prices frequently experience jumps triggered by unpredictable events. In contrast, the path of the GBM is continuous. Despite these simplifications, GBM remains the most prevalently used model for characterizing stock price behavior. This is primarily attributed to its easily computation and its ability to provide reasonably good approximate estimations of stock prices. Then, *S*(*t*) evolves as


dS(t)=S(t)[μdt+σdW(t)],

where σ>0 and μ>r denote the volatility and the appreciation rate, respectively. *W*(*t*) is the standard Brownian motion under measure P, and *W*(*t*) is assumed independent with *Wk*(*t*).

Denote $\pi_{k}(t)$ as the amount invested by insurer *k* in the risky stock at time *t*, and the remainder of its surplus is invested in the risk-free asset. Then under the reinsurance-investment strategy $u_{k}(t):=(q_{k}(t),\pi_{k}(t))$, the dynamics of surplus process $\{{\hat{X}}_{k}^{u_{k}}(t){\}}_{t\in [0,T]}$ of insurer *k* follows

$$d{\hat{X}}_{k}^{u_{k}}(t)=\;[r{\hat{X}}_{k}^{u_{k}}(t)+(\mu -r)\pi_{k}(t)+\lambda_{k}+\eta_{k}q_{k}(t)]dt\;+\sigma \pi_{k}(t)dW(t)+\sigma_{k}q_{k}(t)dW_{k}(t),$$ (2)

${\hat{X}}_{k}^{u_{k}}(0)={\hat{x}}_{k}^{0}$ is insurer *k*’s initial surplus.

### 2.2 Ambiguity attitudes

In the majority of the existing literature on the reinsurance–investment optimization problems, the insurers are assumed to have full faith in the models describing the real-world probability P. Nevertheless, financial and insurance markets are fraught with uncertainties. It is highly debatable which model is truly suitable for depicting the real-world, and even if an appropriate model is selected, accurately estimating the parameters within that model is a formidable challenge. In light of these complexities, following [13] and [18], this paper incorporates ambiguity into the analysis by seeking alternative models, and the model described under the measure P is treated as the reference model. To do this, we define a class of alternative measure $Q_{k}$ which is equivalent to P, as follows


$${𝒬}_{k}:=\{{\mathbb{Q}}_{k}|{\mathbb{Q}}_{k}\sim \mathbb{P}\}.$$

For k∈{1,2}, let $\phi_{k}(t):=(\phi_{k1}(t),\phi_{k2}(t))\in \Phi_{k}$ be $\{{ℱ}_{t}{\}}_{t\in [0,T]}$-progressively measurable processes, where $\Phi_{k}$ is a set of deterministic functions satisfying


$$E^{P}\left[\exp\left(\int_{0}^{T}\frac{\phi_{k1}^{2}(t)+\phi_{k2}^{2}(t)}{2}dt\right)\right]<\infty .$$

Then the Radon–Nikodým derivative process can be defined as:


$$\frac{d{\mathbb{Q}}_{k}}{d\mathbb{P}}{|}_{{ℱ}_{t}}:=\;\Lambda_{k}^{\phi }(t)\;=\exp\{-\int_{0}^{t}\phi_{k1}(s)dW(s)-\frac{1}{2}\int_{0}^{t}(\phi_{k1}(s){)}^{2}ds\;\;\;-\int_{0}^{t}\phi_{k2}(s)dW(s)-\frac{1}{2}\int_{0}^{t}(\phi_{k2}(s){)}^{2}ds\}.$$

Applying the Girsanov’s Theorem, under the probability measure $Q_{k}$, k∈{1,2}, we know that


$$\left\{ \begin{array}{cc} dW^{{\mathbb{Q}}_{k}}(t)= & dW(t)+\phi_{k1}(t),\\ dW_{k}^{{\mathbb{Q}}_{k}}(t)= & dW_{k}(t)+\phi_{k2}(t), \end{array}\right.$$

are standard Brownian motions, and $W^{{\mathbb{Q}}_{k}}(t)$ is independent with $W_{k}^{{\mathbb{Q}}_{k}}(t)$. Accordingly, the wealth process of insurer *k* is given by

$$d{\hat{X}}_{k}^{u_{k}}(t)=\;[r{\hat{X}}_{k}^{u_{k}}(t)+(\mu -r)\pi_{k}(t)+\lambda_{k}+\eta_{k}q_{k}(t)-\sigma \pi_{k}(t)\phi_{k1}(t)\;-\sigma_{k}q_{k}(t)\phi_{k2}(t)]dt+\sigma \pi_{k}(t)dW^{{\mathbb{Q}}_{k}}(t)+\sigma_{k}q_{k}(t)dW_{k}^{{\mathbb{Q}}_{k}}(t).$$ (3)

In financial practice, financial institutions often place greater emphasis on their relative performance compared to their peers. [28] propose an easy handling framework to model the interaction mechanisms between the competition institutions. In this article, we build upon their work and continue to use the relative performance to describe the competition between the two insurers. Specifically, each insurer aims to outperform its competitor in terms of the terminal wealth. Here the relative performance process of insurer *k*, for k,j∈{1,2},k≠j, is defined as


$$X_{k}^{u_{k},u_{j}}(t)=\;(1-n_{k}){\hat{X}}_{k}^{u_{k}}(t)+n_{k}({\hat{X}}_{k}^{u_{k}}(t)-{\hat{X}}_{j(t)}^{u_{j}})\;=\;{\hat{X}}_{k}^{u_{k}}(t)-n_{k}{\hat{X}}_{j}^{u_{j}}(t),$$

where $n_{k}\in [0,1]$ measures the sensitivity of insurer *k* to its competitor insurer *j*’s performance, and the larger the *n*_*k*_, the more the insurer is concerned about increasing its relative surplus, which indicates the game is more competitive.

Under the probability measure $Q_{k}$, the dynamic of insurer *k*’s relative performance is described by

$$dX_{k}^{u_{k},u_{j}}(t)\;=\;[rX_{k}^{u_{k},u_{j}}(t)+(\mu -r)(\pi_{k}(t)-n_{k}\pi_{j}(t))+\lambda_{k}-n_{k}\lambda_{j}+\eta_{k}q_{k}(t)-n_{k}\eta_{j}q_{j}(t)\;-\sigma \pi_{k}(t)\phi_{k1}(t)+n_{k}\sigma \pi_{j}(t)\phi_{j1}(t)-\sigma_{k}q_{k}(t)\phi_{k2}(t)+n_{k}\sigma_{j}q_{j}(t)\phi_{k2}(t)]dt\;+\sigma \pi_{k}(t)dW^{{\mathbb{Q}}_{k}}(t)-n_{k}\sigma \pi_{j}(t)dW^{{\mathbb{Q}}_{j}}(t)+\sigma_{k}q_{k}(t)dW_{k}^{{\mathbb{Q}}_{k}}(t)\;-n_{k}\sigma_{j}q_{j}(t)dW_{j}^{{\mathbb{Q}}_{j}}(t),$$ (4)

with the initial relative performance $X_{k}^{u_{k},u_{j}}(0)=x_{k}^{0}={\hat{x}}_{k}^{0}-n_{k}{\hat{x}}_{j}^{0}$.

### 2.3 The α-robust non-zero-sum game

This section formulates the non-zero-sum game between the two competing insurers under the α-maxmin mean-variance criterion which is formulated in [18]. Following [18] and [20], we develop the α-maxmin mean–variance criterion of insurer *k* as follows: $\forall (t,x_{k})\in [0,T]\times \mathbb{R}$, k,j∈{1,2}, k≠j,

$$J_{k}^{u_{k},u_{j}}(t,x_{k})=\;\alpha_{k}\underset{\phi_{k}\in \Phi_{k}}{\inf}{\underset{―}{J}}_{k}^{\phi_{k},u_{k},u_{j}}(t,x_{k})+{\hat{\alpha }}_{k}\underset{\phi_{k}\in \Phi_{k}}{\sup}{\overset{―}{J}}_{k}^{\phi_{k},u_{k},u_{j}}(t,x_{k}),\;=\;\alpha_{k}{\underset{―}{J}}_{k}^{\underset{―}{\phi_{k}},u_{k},u_{j}}(t,x_{k})+{\hat{\alpha }}_{k}{\overset{―}{J}}_{k}^{\overset{―}{\phi_{k}},u_{k},u_{j}}(t,x_{k}),$$ (5)

where $\alpha_{k}\in [\frac{1}{2},1]$, ${\hat{\alpha }}_{k}=1-\alpha_{k}$,

$${\underset{―}{J}}_{k}^{\phi_{k},u_{k},u_{j}}(t,x_{k})=E_{t,x_{k}}^{\phi_{k}}\left[X_{k}^{u_{k},u_{j}}(T)\right]-\frac{\gamma_{k}}{2}{Var}_{t,x_{k}}^{\phi_{k}}\left[X_{k}^{u_{k},u_{j}}(T)\right]+\int_{t}^{T}h_{\beta }(\phi_{k}(s))ds,$$ (6)

and

$${\overset{―}{J}}_{k}^{\phi_{k},u_{k},u_{j}}(t,x_{k})=E_{t,x_{k}}^{\phi_{k}}\left[X_{k}^{u_{k},u_{j}}(T)\right]-\frac{\gamma_{k}}{2}{Var}_{t,x_{k}}^{\phi_{k}}\left[X_{k}^{u_{k},u_{j}}(T)\right]-\int_{t}^{T}h_{\beta }(\phi_{k}(s))ds,$$ (7)

in which, ${𝔼}_{t,x_{k}}^{\phi_{k}}\left[\cdot \right]:={𝔼}^{{\mathbb{Q}}^{\phi_{k}}}\left[\cdot |X_{k}^{u_{k},u_{j}}(t)=x_{k}\right]$, ${Var}_{t,x_{k}}^{\phi_{k}}\left[\cdot \right]={Var}^{{\mathbb{Q}}^{\phi_{k}}}\left[\cdot |X_{k}^{u_{k},u_{j}}(t)=x_{k}\right]$. And $\underset{―}{\phi_{k}}$ and $\overset{―}{\phi_{k}}$ are the probability distortion functions to achieve the infimum and supremum in Eq (5), respectively, and the penalty function which penalizes the deviation of alternative measure ℚ from the reference measure P is selected as $h_{\beta }(\phi_{k}(s))=\frac{\phi_{k1}^{2}}{2\beta_{k1}}+\frac{\phi_{k2}^{2}}{2\beta_{k2}}$.

In Eq (5), there are four parameters, $\alpha_{k}$, $\gamma_{k}$, and $\beta_{ki}$, i∈{1,2}. First, $\alpha_{k}$ represents insurer *k*’s ambiguity attitude, and insurer *k* with a larger $\alpha_{k}$ is more ambiguity aversion. In particular, $\alpha_{k}=\frac{1}{2},1$ represent the ambiguity-neutral, and extremely ambiguity-averse attitude, respectively. Second, $\gamma_{k}$ is the insurer *k*’s risk aversion coefficient. Third, $\beta_{ki}$, i∈{1,2}, are the ambiguity aversion coefficients which measure insurer *k*’s level of ambiguity towards the reference measure.

The two insurers aim to identify an optimal strategy that maximizes the expression in Eq (5). Under this optimization criterion in place, the strategic interaction is defined as follows:

**Problem 2.1** The non-zero-sum stochastic differential game between the two competing insurers, under the α-maxmin mean-variance criterion, involves the search for Nash equilibrium strategies $(u_{1}^{*},u_{2}^{*})\in {𝒰}_{1}\times {𝒰}_{2}$ such that for any $(u_{1},u_{2})\in {𝒰}_{1}\times {𝒰}_{2}$, we have

$$J_{1}^{u_{1}^{*},u_{2}^{*}}(t,x_{1})\geq J_{1}^{u_{1},u_{2}^{*}}(t,x_{1}),\;J_{2}^{u_{1}^{*},u_{2}^{*}}(t,x_{2})\geq J_{2}^{u_{1}^{*},u_{2}}(t,x_{2}).$$ (8)

And the admissible strategy is defined below.

**Definition 2.2** (*Admissible Strategy*) For any given t∈[0,T], a strategy $u_{k}(t):=(q_{k}(t),\pi_{k}(t))$, k∈{1,2}, is said to be admissible, if it satisfies:

(i) ∀t∈[0,T], *u*_*k*_(*t*) is ${ℱ}_{t}$-measurable, $q_{k}(t)\geq 0$;

(ii) $\forall (x_{k},t)\in \mathbb{R}\;\times \;[0,T]$, it holds that ${𝔼}^{\phi_{k}}\left[\int_{0}^{T}{\left\|u_{k}(s)\right\|}^{2}ds\right]<\infty$ for any $\phi_{k}\in \Phi_{k}$, where ${\left\|u_{k}(s)\right\|}^{2}=q_{k}^{2}(s)+\pi_{k}^{2}(s)$;

(iii) $\forall (x_{k},t)\in \mathbb{R}\times [0,T]$, Eq (3) has a unique strong solution.

Let ${𝒰}_{k}$ denote the set of all admissible strategies for insurer *k*.

In order to handle the time-inconsistent issue in Problem 2.1, following [29], we give the definition of equilibrium strategy as follows:

**Definition 2.3** Let $u_{k}^{*}=(\cdot ,q_{k}^{*}(\cdot ),\pi_{k}^{*}(\cdot ))$ be an admissible strategy of insurer *k*. For any initial state $(t,x_{k})\in [0,T]\times R$ and ϵ>0, when its competitor’s optimal strategy $\pi_{j}^{*}$ is known, we define a perturbed strategy $u_{k}^{\epsilon }$ as follows:


$$u_{k}^{\epsilon }(v):=\left\{ \begin{array}{cc} {\hat{u}}_{k}(v), & v\in [t,t+\epsilon ),\\ u_{k}^{*}(v), & v\in [t+\epsilon ,T]. \end{array}\right.$$

where ${\hat{u}}_{k}\in {𝒰}_{k}$. If $\forall {\hat{u}}_{k}\in {𝒰}_{k}$, we have


$$\lim_{\epsilon ↓0}\inf\frac{J_{k}^{u_{k}^{*},u_{j}^{*}}(t,x_{k})-J_{k}^{u_{k}^{\epsilon },u_{j}^{*}}(t,x_{k})}{\epsilon }\geq 0,$$

then $u_{k}^{*}(\cdot )$ is called an equilibrium strategy of insurer *k*, and its equilibrium value function is given by $J_{k}^{u_{k}^{*},u_{j}^{*}}(t,x_{k})$.

According to Definition 2.3, the equilibrium strategy is time-consistent.

## 3 Main results

This section presents the verification theorem, followed by the derivation of the α-robust equilibrium strategy. We first denote


$$C^{1,2}([0,T]\times \mathbb{R}):=\{\phi (t,x)|\phi (t,x)\text{is continuously differentiable for}\;t\in [0,T]\;\text{and twice continuously differentiable for}\;x\in \mathbb{R}\},$$

and for any $(t,x_{k})\in [0,t]\times \mathbb{R}$, $\psi_{k}(t,x_{k})\in C^{1,2}([0,T]\times \mathbb{R})$, we define an infinitesimal operator as

$${ℒ}^{\phi_{k},\phi_{j},u_{k},u_{j}}\psi_{k}(t,x_{k})\;=\;\frac{\partial \psi_{k}(t,x_{k})}{\partial t}+[rx_{k}+(\mu -r)(\pi_{k}-n_{k}\pi_{j})+\lambda_{k}-n_{k}\lambda_{j}+\eta_{k}q_{k}-n_{k}\eta_{j}q_{j}-\sigma \pi_{k}\phi_{k1}\;+n_{k}\sigma \pi_{j}\phi_{j1}-\sigma_{k}q_{k}\phi_{k2}+n_{k}\sigma_{j}q_{j}\phi_{j2}]\frac{\partial \psi_{k}(t,x_{k})}{\partial x_{k}}+\frac{1}{2}(\sigma^{2}\pi_{k}^{2}+n_{k}^{2}\sigma^{2}\pi_{j}^{2}-2n_{k}\sigma^{2}\pi_{k}\pi_{j}\;+\sigma_{k}^{2}q_{k}^{2}+n_{k}^{2}\sigma_{j}^{2}q_{j}^{2}-2\rho n_{k}\sigma_{k}\sigma_{j}q_{k}q_{j})\frac{\partial^{2}\psi_{k}(t,x_{k})}{\partial x_{k}^{2}}.$$ (9)

Next we present the main results for the two competing insurers.

**Theorem 3.1.** (*Verification Theorem*) For Problem 2.1, if there are real-valued functions $V_{k}(t,x_{k}),{\underset{―}{g}}_{k}(t,x_{k}),{\overset{―}{g}}_{k}(t,x_{k})\in C^{1,2}([0,T]\times \mathbb{R})$ satisfy the following conditions:

(1) For any $(t,x_{k})\in [0,T]\times \mathbb{R}$,

$$\underset{u_{k}\in {𝒰}_{k}}{\sup}\{\alpha_{k}\underset{\phi_{k}\in \Phi_{k}}{\inf}[{ℒ}^{u_{k},u_{j}^{*},\phi_{k},{\underset{―}{\phi }}_{j}^{*}}\;V_{k}(t,x_{k})-\frac{\gamma_{k}}{2}{ℒ}^{u_{k},u_{j}^{*},\phi_{k},{\underset{―}{\phi }}_{j}^{*}}{\underset{―}{g}}_{k}^{2}(t,x_{k})\;+\gamma_{k}{\underset{―}{g}}_{k}(t,x_{k}){ℒ}^{u_{k},u_{j}^{*},\phi_{k},{\underset{―}{\phi }}_{j}^{*}}{\underset{―}{g}}_{k}(t,x_{k})+h_{\beta }(\phi_{k})]\;+{\hat{\alpha }}_{k}\underset{\phi_{k}\in \Phi_{k}}{\sup}[{ℒ}^{u_{k},u_{j}^{*},\phi_{k},{\overset{―}{\phi }}_{j}^{*}}V_{k}(t,x_{k})-\frac{\gamma_{k}}{2}{ℒ}^{u_{k},u_{j}^{*},\phi_{k},{\overset{―}{\phi }}_{j}^{*}}{\overset{―}{g}}_{k}^{2}(t,x_{k})\;+\gamma_{k}{\underset{―}{g}}_{k}(t,x_{k}){ℒ}^{u_{k},u_{j}^{*},\phi_{k},{\overset{―}{\phi }}_{j}^{*}}{\overset{―}{g}}_{k}(t,x_{k})-h_{\beta }(\phi_{k})]\}=0,$$ (10)

(2) For any $(t,x_{k})\in [0,T]\times \mathbb{R}$,

$$\left\{ \begin{array}{c} V_{k}(T,x_{k})=x_{k},\\ {ℒ}^{u_{k}^{*},u_{j}^{*},{\underset{―}{\phi }}_{k}^{*},{\underset{―}{\phi }}_{j}^{*}}{\underset{―}{g}}_{k}(t,x_{k})={ℒ}^{u_{k}^{*},u_{j}^{*},{\overset{―}{\phi }}_{k}^{*},{\overset{―}{\phi }}_{j}^{*}}{\overset{―}{g}}_{k}(t,x_{k})=0,\\ {\underset{―}{g}}_{k}(T,x_{k})={\overset{―}{g}}_{k}(T,x_{k})=x_{k}. \end{array}\right.$$ (11)

(3) For any $(t,x_{k})\in [0,T]\times \mathbb{R}$, $u_{k}^{*}(t)$, ${\underset{―}{\phi }}_{k}^{*}(t)$, ${\overset{―}{\phi }}_{k}^{*}(t)$, ${ℒ}^{u_{k}^{*},u_{j}^{*},{\underset{―}{\phi }}_{k}^{*},{\underset{―}{\phi }}_{j}^{*}}V(t,x_{k})$, ${ℒ}^{u_{k}^{*},u_{j}^{*},{\overset{―}{\phi }}_{k}^{*},{\overset{―}{\phi }}_{j}^{*}}V(t,x_{k})$, ${ℒ}^{u_{k}^{*},u_{j}^{*},{\underset{―}{\phi }}_{k}^{*},{\underset{―}{\phi }}_{j}^{*}}{\underset{―}{g}}_{k}^{2}(t,x_{k})$ and ${ℒ}^{u_{k}^{*},u_{j}^{*},{\overset{―}{\phi }}_{k}^{*},{\overset{―}{\phi }}_{j}^{*}}{\overset{―}{g}}_{k}^{2}(t,x_{k})$ are deterministic functions of *t* and independent of *x*_*k*_.

(4) ${\underset{―}{\phi }}_{k}^{*}={\underset{―}{\phi }}_{k}^{u_{k}^{*}}$ and ${\overset{―}{\phi }}_{k}^{*}={\overset{―}{\phi }}_{k}^{u_{k}^{*}}$.

Then $u_{k}^{*}$ is the α-robust equilibrium strategy, $V_{k}(t,x_{k})=J_{k}^{u_{k}^{*},u_{j}^{*}}(t,x_{k})$ is the equilibrium value function of insurer *k*. Besides, ${\underset{―}{g}}_{k}(t,x_{k})=E_{t,x_{k}}^{{\underset{―}{\phi }}_{k}^{*}}[X_{k}^{u_{k}^{*},u_{j}^{*}}(T)]$, ${\overset{―}{g}}_{k}(t,x_{k})=E_{t,x_{k}}^{{\overset{―}{\phi }}_{k}^{*}}[X_{k}^{u_{k}^{*},u_{j}^{*}}(T)]$.

*Proof*: See S1 Appendix. ◻

**Theorem 3.2.** Consider the α-robust game between the two competing insurers in Problems 2.1.

(1) The time-consistent optimal reinsurance strategy of insurer *k*, k∈{1,2}, is given by:

$$q_{k}^{*}=\frac{\eta_{k}\sigma_{j}[\gamma_{j}-({\hat{\alpha }}_{j}-\alpha_{j})\beta_{j2}]+n_{k}\rho \eta_{j}\sigma_{k}\gamma_{k}}{\{[\gamma_{k}-({\hat{\alpha }}_{k}-\alpha_{k})\beta_{k2}][\gamma_{j}-({\hat{\alpha }}_{j}-\alpha_{j})\beta_{j2}]-n_{k}n_{j}\rho^{2}\gamma_{k}\gamma_{j}\}\sigma_{k}^{2}\sigma_{j}e^{r(T-t)}},$$ (12)

and the time-consistent optimal investment strategy is given by:

$$\pi_{k}^{*}=\frac{\left(\mu -r)[\gamma_{j}-({\hat{\alpha }}_{j}-\alpha_{j})\beta_{j1}+n_{k}\gamma_{k}\right]}{\{[\gamma_{k}-({\hat{\alpha }}_{k}-\alpha_{k})\beta_{k1}][\gamma_{j}-({\hat{\alpha }}_{j}-\alpha_{j})\beta_{j1}]-n_{k}n_{j}\gamma_{k}\gamma_{j}\}\sigma^{2}e^{r(T-t)}}.$$ (13)

(2) The equilibrium value function is given by

$$V_{k}(t,x_{k})=e^{r(T-t)}x_{k}+\frac{n_{k}\lambda_{j}-\lambda_{k}}{r}(1-e^{r(T-t)})+\int_{t}^{T}d_{k1}(s)ds+\int_{t}^{T}d_{k2}(s)ds,$$ (14)

where


$$\left\{ \begin{array}{cc} d_{k1}(s)= & \frac{{\hat{\alpha }}_{k}-\alpha_{k}}{2}\beta_{k2}\sigma_{k}^{2}(q_{k}^{*}{)}^{2}e^{2r(T-s)}+(\alpha_{k}-{\hat{\alpha }}_{k})n_{k}\beta_{j2}\sigma_{j}^{2}(q_{j}^{*}{)}^{2}e^{2r(T-s)}\\ & -[\sigma_{k}^{2}(q_{k}^{*}{)}^{2}+n_{k}^{2}\sigma_{j}^{2}(q_{j}^{*}{)}^{2}-2\rho n_{k}\sigma_{k}\sigma_{j}q_{k}^{*}q_{j}^{*}]\frac{(\alpha +\hat{\alpha })\gamma_{k}}{2}e^{2r(T-s)}\\ & +(\eta_{k}q_{k}^{*}-n_{k}\eta_{j}q_{j}^{*})e^{r(T-s)}\\ d_{k2}(s)= & \frac{{\hat{\alpha }}_{k}-\alpha_{k}}{2}\beta_{k1}\sigma^{2}(\pi_{k}^{*}{)}^{2}e^{2r(T-s)}+(\alpha_{k}-{\hat{\alpha }}_{k})n_{k}\beta_{j1}\sigma^{2}(\pi_{j}^{*}{)}^{2}e^{2r(T-s)}\\ & -\frac{(\alpha_{k}+{\hat{\alpha }}_{k})\gamma_{k}}{2}e^{2r(T-s)}[\sigma^{2}(\pi_{k}^{*}{)}^{2}+n_{k}^{2}\sigma^{2}(\pi_{j}^{*}{)}^{2}-2n_{k}\sigma^{2}\pi_{k}^{*}\pi_{j}^{*}]\\ & +(\mu -r)(\pi_{k}^{*}-n_{k}\pi_{j}^{*})e^{r(T-s)}. \end{array}\right.$$

(3) The associated probability distortion functions of extremely ambiguity-averse measure and the extremely ambiguity-seeking measure are given respectively by

$$\left\{ \begin{array}{c} {\underset{―}{\phi }}_{k1}^{*}=\beta_{k1}\sigma \pi_{k}^{*}e^{r(T-t)},\\ {\underset{―}{\phi }}_{k2}^{*}=\beta_{k2}\sigma_{k}q_{k}^{*}e^{r(T-t)}, \end{array}\right.$$ (15)

and

$$\left\{ \begin{array}{c} {\overset{―}{\phi }}_{k1}^{*}=-\beta_{k1}\sigma \pi_{k}^{*}e^{r(T-t)},\\ {\overset{―}{\phi }}_{k2}^{*}=-\beta_{k2}\sigma_{k}q_{k}^{*}e^{r(T-t)}. \end{array}\right.$$ (16)

*Proof*: Assume $V_{k}(t,x_{k})$, ${\underset{―}{g}}_{k}(t,x_{k})$, ${\overset{―}{g}}_{k}(t,x_{k})$, ${\underset{―}{\phi }}^{u_{k}^{*}}$, ${\overset{―}{\phi }}^{u_{k}^{*}}$ satisfying condition (1) in Theorem 3.1. Through simple calculations, we obtain

$$0=\underset{u_{k}\in {𝒰}_{k}}{\sup}\{\frac{\partial V_{k}(t,x_{k})}{\partial t}+[rx_{k}+(\mu -r)(\pi_{k}-n_{k}\pi_{j}^{*})+\lambda_{k}-n_{k}\lambda_{j}+\eta_{k}q_{k}-n_{k}\eta_{j}q_{j}^{*}]\frac{\partial V_{k}(t,x_{k})}{\partial x_{k}}\;+\frac{1}{2}(\sigma^{2}\pi_{k}^{2}+n_{k}^{2}\sigma^{2}(\pi_{j}^{*}{)}^{2}-2n_{k}\sigma^{2}\pi_{k}\pi_{j}^{*}+\sigma_{k}^{2}q_{k}^{2}+n_{k}^{2}\sigma_{j}^{2}(q_{j}^{*}{)}^{2}-2\rho n_{k}\sigma_{k}\sigma_{j}q_{k}q_{j}^{*})\;\times (\frac{\partial^{2}W_{k}(t,x_{k})}{\partial x_{k}^{2}}-\alpha_{k}\gamma_{k}(\frac{\partial {\underset{―}{g}}_{k}(t,x_{k})}{\partial x_{k}}{)}^{2}-{\hat{\alpha }}_{k}\gamma_{k}(\frac{\partial {\overset{―}{g}}_{k}(t,x_{k})}{\partial x_{k}}{)}^{2})\;+\alpha_{k}\underset{\phi_{k}\in \Phi_{k}}{\inf}\{\left(-\sigma \pi_{k}\phi_{k1}+n_{k}\sigma \pi_{j}^{*}{\underset{―}{\phi }}_{j1}^{*}-\sigma_{k}q_{k}\phi_{k2}+n_{k}\sigma_{j}q_{j}^{*}{\underset{―}{\phi }}_{j2}^{*}\right)\frac{\partial V_{k}(t,x_{k})}{\partial x_{k}}+\frac{\phi_{k1}^{2}}{2\beta_{k1}}\;+\frac{\phi_{k2}^{2}}{2\beta_{k2}}\}+{\hat{\alpha }}_{k}\underset{\phi_{k}\in \Phi_{k}}{\sup}\{\left(-\sigma \pi_{k}\phi_{k1}+n_{k}\sigma \pi_{j}^{*}{\overset{―}{\phi }}_{j1}^{*}-\sigma_{k}q_{k}\phi_{k2}+n_{k}\sigma_{j}q_{j}^{*}{\overset{―}{\phi }}_{j2}^{*}\right)\frac{\partial V_{k}(t,x_{k})}{\partial x_{k}}\;-\frac{\phi_{k1}^{2}}{2\beta_{k1}}-\frac{\phi_{k2}^{2}}{2\beta_{k2}}\}\}.$$ (17)

In order to solve (11) and (17), we assume that the forms of the solutions are as follows:

$$\left\{ \begin{array}{c} V_{k}(t,x_{k})=A_{k}(t)x_{k}+B_{k}(t),\\ {\underset{―}{g}}_{k}(t,x_{k})={\underset{―}{a}}_{k}(t)x_{k}+{\underset{―}{b}}_{k}(t),\\ {\overset{―}{g}}_{k}(t,x_{k})={\overset{―}{a}}_{k}(t)x_{k}+{\overset{―}{b}}_{k}(t), \end{array}\right.$$ (18)

where *A*_*k*_(*t*), *B*_*k*_(*t*), ${\underset{―}{a}}_{k}(t)$, ${\underset{―}{b}}_{k}(t)$, ${\overset{―}{a}}_{k}(t)$, ${\overset{―}{b}}_{k}(t)$ are functions of *t*. By the first and the third relation of (11), we have the boundary conditions


$$\left\{ \begin{array}{c} A_{k}(T)={\underset{―}{a}}_{k}(T)={\overset{―}{a}}_{k}(T)=1,\\ B_{k}(T)={\underset{―}{b}}_{k}(T)={\overset{―}{b}}_{k}(T)=0. \end{array}\right.$$

Substituting (18) and their partial derivatives into (17) yields

$$0=\underset{u_{k}\in {𝒰}_{k}}{\sup}\{A_{k}^{\prime }x_{k}+B_{k}^{\prime }+[rx_{k}+(\mu -r)(\pi_{k}-n_{k}\pi_{j}^{*})+\lambda_{k}-n_{k}\lambda_{j}+\eta_{k}q_{k}-n_{k}\eta_{j}q_{j}^{*}]A_{k}\;+\frac{1}{2}(\sigma^{2}\pi_{k}^{2}+n_{k}^{2}\sigma^{2}(\pi_{j}^{*}{)}^{2}-2n_{k}\sigma^{2}\pi_{k}\pi_{j}^{*}+\sigma_{k}^{2}q_{k}^{2}+n_{k}^{2}\sigma_{j}^{2}(q_{j}^{*}{)}^{2}-2\rho n_{k}\sigma_{k}\sigma_{j}q_{k}q_{j}^{*})\;\times (-\alpha_{k}\gamma_{k}{\underset{―}{a}}_{k}^{2}-{\hat{\alpha }}_{k}\gamma_{k}{\overset{―}{a}}_{k}^{2})\;+\alpha_{k}\underset{\phi_{k}\in \Phi_{k}}{\inf}\{(-\sigma \pi_{k}\phi_{k1}+n_{k}\sigma \pi_{j}^{*}{\underset{―}{\phi }}_{j1}^{*}-\sigma_{k}q_{k}\phi_{k2}+n_{k}\sigma_{j}q_{j}^{*}{\underset{―}{\phi }}_{j2}^{*})A_{k}+\frac{\phi_{k1}^{2}}{2\beta_{k1}}+\frac{\phi_{k2}^{2}}{2\beta_{k2}}\}\;+{\hat{\alpha }}_{k}\underset{\phi_{k}\in \Phi_{k}}{\sup}\{(-\sigma \pi_{k}\phi_{k1}+n_{k}\sigma \pi_{j}^{*}{\overset{―}{\phi }}_{j1}^{*}-\sigma_{k}q_{k}\phi_{k2}+n_{k}\sigma_{j}q_{j}^{*}{\overset{―}{\phi }}_{j2}^{*})A_{k}-\frac{\phi_{k1}^{2}}{2\beta_{k1}}-\frac{\phi_{k2}^{2}}{2\beta_{k2}}\}\}.$$ (19)

By the first-order condition on Eq (19) with respect to $\phi_{k}$, the infimum and the supremum of $\phi_{k}$ in Eq (19) can be achieved respectively at

$$\left\{ \begin{array}{c} {\underset{―}{\phi }}_{k1}^{*}=\beta_{k1}\sigma \pi_{k}A_{k},{\underset{―}{\phi }}_{k2}^{*}=\beta_{k2}\sigma_{k}q_{k}A_{k},\\ {\overset{―}{\phi }}_{k1}^{*}=-\beta_{k1}\sigma \pi_{k}A_{k},{\overset{―}{\phi }}_{k2}^{*}=-\beta_{k2}\sigma_{k}q_{k}A_{k}. \end{array}\right.$$ (20)

Inserting Eq (20) back into Eq (19) yields

$$0=\underset{u_{k}\in {𝒰}_{k}}{\sup}\{A_{k}^{\prime }x_{k}+B_{k}^{\prime }+[rx_{k}+(\mu -r)(\pi_{k}-n_{k}\pi_{j}^{*})+\lambda_{k}-n_{k}\lambda_{j}+\eta_{k}q_{k}-n_{k}\eta_{j}q_{j}^{*}]A_{k}\;+\frac{1}{2}(\sigma^{2}\pi_{k}^{2}+n_{k}^{2}\sigma^{2}(\pi_{j}^{*}{)}^{2}-2n_{k}\sigma^{2}\pi_{k}\pi_{j}^{*}+\sigma_{k}^{2}q_{k}^{2}+n_{k}^{2}\sigma_{j}^{2}(q_{j}^{*}{)}^{2}-2\rho n_{k}\sigma_{k}\sigma_{j}q_{k}q_{j}^{*})\;\times (-\alpha \gamma_{k}{\underset{―}{a}}_{k}^{2}-\hat{\alpha }\gamma_{k}{\overset{―}{a}}_{k}^{2})+\frac{1}{2}({\hat{\alpha }}_{k}-\alpha_{k})(\beta_{k1}\sigma^{2}\pi_{k}^{2}+\beta_{k2}\sigma_{k}^{2}q_{k}^{2})A_{k}^{2}\;+\alpha_{k}n_{k}\beta_{j1}\sigma^{2}(\pi_{j}^{*}{)}^{2}A_{j}A_{k}+\alpha_{k}n_{k}\beta_{j2}\sigma_{j}^{2}(q_{j}^{*}{)}^{2}A_{j}A_{k}-{\hat{\alpha }}_{k}n_{k}\beta_{j1}\sigma^{2}(\pi_{j}^{*}{)}^{2}A_{j}A_{k}\;-{\hat{\alpha }}_{k}n_{k}\beta_{j2}\sigma_{j}^{2}(q_{j}^{*}{)}^{2}A_{j}A_{k}\}.$$ (21)

Furthermore, applying the first-order condition on Eq (21) with respect to *q*_*k*_ and $\pi_{k}$ gives

$$q_{k}^{*}=\frac{-\eta_{k}A_{k}+\rho n_{k}\sigma_{k}\sigma_{j}\left(-\alpha_{k}\gamma_{k}{\underset{―}{a}}_{k}^{2}-{\hat{\alpha }}_{k}\gamma_{k}{\overset{―}{a}}_{k}^{2}\right)q_{j}^{*}}{\sigma_{k}^{2}\left[-\alpha_{k}\gamma_{k}{\underset{―}{a}}_{k}^{2}-{\hat{\alpha }}_{k}\gamma_{k}{\overset{―}{a}}_{k}^{2}+({\hat{\alpha }}_{k}-\alpha_{k})\beta_{k2}A_{k}^{2}\right]},$$ (22)

and

$$\pi_{k}^{*}=\frac{-(\mu -r)A_{k}+n_{k}\sigma^{2}\left(-\alpha_{k}\gamma_{k}{\underset{―}{a}}_{k}^{2}-{\hat{\alpha }}_{k}\gamma_{k}{\overset{―}{a}}_{k}^{2}\right)\pi_{j}^{*}}{\sigma^{2}\left[-\alpha_{k}\gamma_{k}{\underset{―}{a}}_{k}^{2}-{\hat{\alpha }}_{k}\gamma_{k}{\overset{―}{a}}_{k}^{2}+({\hat{\alpha }}_{k}-\alpha_{k})\beta_{k1}A_{k}^{2}\right]}.$$ (23)

Substituting Eq (22) and (23) back into (21) and the second equation of (11), we obtain

$$\left\{ \begin{array}{c} A_{k}^{\prime }x_{k}+B_{k}^{\prime }+[rx_{k}+(\mu -r)(\pi_{k}^{*}-n_{k}\pi_{j}^{*})+\lambda_{k}-n_{k}\lambda_{j}+\eta_{k}q_{k}^{*}-n_{k}\eta_{j}q_{j}^{*}]A_{k}\\ \;+\frac{1}{2}(\sigma^{2}(\pi_{k}^{*}{)}^{2}+n_{k}^{2}\sigma^{2}(\pi_{j}^{*}{)}^{2}-2n_{k}\sigma^{2}\pi_{k}^{*}\pi_{j}^{*}+\sigma_{k}^{2}(q_{k}^{*}{)}^{2}+n_{k}^{2}\sigma_{j}^{2}(q_{j}^{*}{)}^{2}-2\rho n_{k}\sigma_{k}\sigma_{j}q_{k}^{*}q_{j}^{*})\\ \;\times (-\alpha_{k}\gamma_{k}{\underset{―}{a}}_{k}^{2}-{\hat{\alpha }}_{k}\gamma_{k}{\overset{―}{a}}_{k}^{2})+\frac{1}{2}({\hat{\alpha }}_{k}-\alpha_{k})(\beta_{k1}\sigma^{2}(\pi_{k}^{*}{)}^{2}+\beta_{k2}\sigma_{k}^{2}(q_{k}^{*}{)}^{2})A_{k}^{2}\\ \;+\alpha_{k}n_{k}\beta_{j1}\sigma^{2}(\pi_{j}^{*}{)}^{2}A_{j}A_{k}+\alpha n_{k}\beta_{j2}\sigma_{j}^{2}(q_{j}^{*}{)}^{2}A_{j}A_{k}-{\hat{\alpha }}_{k}n_{k}\beta_{j1}\sigma^{2}(\pi_{j}^{*}{)}^{2}A_{j}A_{k}\\ \;-{\hat{\alpha }}_{k}n_{k}\beta_{j2}\sigma_{j}^{2}(q_{j}^{*}{)}^{2}A_{j}A_{k}=0,\\ {\underset{―}{a}}_{k}^{\prime }x_{k}+{\underset{―}{b}}_{k}^{\prime }+[rx_{k}+(\mu -r)(\pi_{k}^{*}-n_{k}\pi_{j}^{*})+\lambda_{k}-n_{k}\lambda_{j}+\eta_{k}q_{k}^{*}-n_{k}\eta_{j}q_{j}^{*}-\beta_{k1}\sigma^{2}(\pi_{k}^{*}{)}^{2}A_{k}\\ \;+n_{k}\beta_{j1}\sigma^{2}(\pi_{j}^{*}{)}^{2}A_{j}-\beta_{k2}\sigma_{k}^{2}(q_{k}^{*}{)}^{2}A_{k}+n_{k}\beta_{j2}\sigma_{j}^{2}(q_{j}^{*}{)}^{2}]A_{j}A_{k}=0,\\ {\overset{―}{a}}_{k}^{\prime }x_{k}+{\overset{―}{b}}_{k}^{\prime }+[rx_{k}+(\mu -r)(\pi_{k}^{*}-n_{k}\pi_{j}^{*})+\lambda_{k}-n_{k}\lambda_{j}+\eta_{k}q_{k}^{*}-n_{k}\eta_{j}q_{j}^{*}-\beta_{k1}\sigma^{2}(\pi_{k}^{*}{)}^{2}A_{k}\\ \;-n_{k}\beta_{j1}\sigma^{2}(\pi_{j}^{*}{)}^{2}A_{j}-\beta_{k2}\sigma_{k}^{2}(q_{k}^{*}{)}^{2}A_{k}-n_{k}\beta_{j2}\sigma_{j}^{2}(q_{j}^{*}{)}^{2}A_{j}]A_{k}=0. \end{array}\right.$$ (24)

By matching the coefficients of the terms of *x*_*k*_, we have


$$A_{k}^{\prime }(t)+rA_{k}(t)={\underset{―}{a}}_{k}^{\prime }(t)+r{\underset{―}{a}}_{k}(t)={\overset{―}{a}}_{k}^{\prime }(t)+r{\overset{―}{a}}_{k}(t)=0,$$

Combining with $A_{k}(T)={\underset{―}{a}}_{k}(T)={\overset{―}{a}}_{k}(T)=1$ yields

$$A_{k}(t)={\underset{―}{a}}_{k}(t)={\overset{―}{a}}_{k}(t)=e^{r(T-t)}.$$ (25)

Moreover, the time-consistent equilibrium strategy of insurer *k* satisfies

$$\left\{ \begin{array}{c} q_{k}^{*}=\frac{-\eta_{k}A_{k}+\rho n_{k}\sigma_{k}\sigma_{j}\left(-\alpha \gamma_{k}{\underset{―}{a}}_{k}^{2}-\hat{\alpha }\gamma_{k}{\overset{―}{a}}_{k}^{2}\right)q_{j}^{*}}{\sigma_{k}^{2}\left[-\alpha \gamma_{k}{\underset{―}{a}}_{k}^{2}-\hat{\alpha }\gamma_{k}{\overset{―}{a}}_{k}^{2}+(\hat{\alpha }-\alpha )\beta_{k2}A_{k}^{2}\right]}=\frac{\eta_{k}+\rho n_{k}\sigma_{k}\sigma_{j}\gamma_{k}q_{j}^{*}e^{r(T-t)}}{\left[\gamma_{k}-({\hat{\alpha }}_{k}-\alpha_{k})\beta_{k2}\right]\sigma_{k}^{2}e^{r(T-t)}},\\ \pi_{k}^{*}=\frac{-(\mu -r)A_{k}+n_{k}\sigma^{2}\left(-\alpha \gamma_{k}{\underset{―}{a}}_{k}^{2}-\hat{\alpha }\gamma_{k}{\overset{―}{a}}_{k}^{2}\right)\pi_{j}^{*}}{\sigma^{2}\left[-\alpha \gamma_{k}{\underset{―}{a}}_{k}^{2}-\hat{\alpha }\gamma_{k}{\overset{―}{a}}_{k}^{2}+(\hat{\alpha }-\alpha )\beta_{k1}A_{k}^{2}\right]}=\frac{(\mu -r)+n_{k}\sigma^{2}\gamma_{k}\pi_{j}^{*}e^{r(T-t)}}{\left[\gamma_{k}-({\hat{\alpha }}_{k}-\alpha_{k})\beta_{k1}\right]\sigma^{2}e^{r(T-t)}}. \end{array}\right.$$ (26)

Then we can obtain Eq (12) which is the reinsurance strategy of each insurer by solving the system of equations:


$$\left\{ \begin{array}{c} q_{1}^{*}=\frac{\eta_{1}+\rho n_{1}\sigma_{1}\sigma_{2}\gamma_{1}q_{2}^{*}e^{r(T-t)}}{\left[\gamma_{1}-({\hat{\alpha }}_{1}-\alpha_{1})\beta_{12}\right]\sigma_{1}^{2}e^{r(T-t)}},\\ q_{2}^{*}=\frac{\eta_{2}+\rho n_{2}\sigma_{2}\sigma_{1}\gamma_{2}q_{1}^{*}e^{r(T-t)}}{\left[\gamma_{2}-({\hat{\alpha }}_{2}-\alpha_{2})\beta_{22}\right]\sigma_{2}^{2}e^{r(T-t)}}, \end{array}\right.$$

and (13) which is the investment strategy of each insurer by solving the system of equations:


$$\left\{ \begin{array}{c} \pi_{1}^{*}=\frac{(\mu -r)+n_{1}\sigma^{2}\gamma_{1}\pi_{2}^{*}e^{r(T-t)}}{\left[\gamma_{1}-({\hat{\alpha }}_{1}-\alpha_{1})\beta_{11}\right]\sigma^{2}e^{r(T-t)}},\\ \pi_{2}^{*}=\frac{(\mu -r)+n_{2}\sigma^{2}\gamma_{2}\pi_{1}^{*}e^{r(T-t)}}{\left[\gamma_{2}-({\hat{\alpha }}_{2}-\alpha_{2})\beta_{21}\right]\sigma^{2}e^{r(T-t)}}. \end{array}\right.$$

Then substituting Eq (25) back into (24), combining with $B_{k}(T)={\underset{―}{b}}_{k}(T)={\overset{―}{b}}_{k}(T)=0$, we yield

$$B_{k}(t)=\frac{n_{k}\lambda_{j}-\lambda_{k}}{r}(1-e^{r(T-t)})+\int_{t}^{T}d_{k1}(s)ds+\int_{t}^{T}d_{k2}(s)ds,$$ (27)

$${\underset{―}{b}}_{k}(t)=\frac{n_{k}\lambda_{j}-\lambda_{k}}{r}(1-e^{r(T-t)})+\int_{t}^{T}{\underset{―}{d}}_{k1}(s)ds+\int_{t}^{T}{\underset{―}{d}}_{k2}(s)ds,$$ (28)

$${\overset{―}{b}}_{k}(t)=\frac{n_{k}\lambda_{j}-\lambda_{k}}{r}(1-e^{r(T-t)})+\int_{t}^{T}{\overset{―}{d}}_{k1}(s)ds+\int_{t}^{T}{\overset{―}{d}}_{k2}(s)ds,$$ (29)

where


$$d_{k1}(s)\;=\;[\eta_{k}q_{k}^{*}-n_{k}\eta_{j}q_{j}^{*}+\frac{{\hat{\alpha }}_{k}-\alpha_{k}}{2}\beta_{k2}\sigma_{k}^{2}(q_{k}^{*}{)}^{2}e^{r(T-s)}+(\alpha_{k}-{\hat{\alpha }}_{k})n_{k}\beta_{j2}\sigma_{j}^{2}(q_{j}^{*}{)}^{2}e^{r(T-s)}]e^{r(T-s)}\;-\frac{(\alpha +\hat{\alpha })\gamma_{k}}{2}e^{2r(T-s)}[\sigma_{k}^{2}(q_{k}^{*}{)}^{2}+n_{k}^{2}\sigma_{j}^{2}(q_{j}^{*}{)}^{2}-2\rho n_{k}\sigma_{k}\sigma_{j}q_{k}^{*}q_{j}^{*}],\;d_{k2}(s)\;=\;[(\mu -r)(\pi_{k}^{*}-n_{k}\pi_{j}^{*})+\frac{{\hat{\alpha }}_{k}-\alpha_{k}}{2}\beta_{k1}\sigma^{2}(\pi_{k}^{*}{)}^{2}e^{r(T-s)}+(\alpha_{k}-{\hat{\alpha }}_{k})n_{k}\beta_{j1}\sigma^{2}(\pi_{j}^{*}{)}^{2}e^{r(T-s)}]\;\times e^{r(T-s)}-\frac{(\alpha_{k}+{\hat{\alpha }}_{k})\gamma_{k}}{2}e^{2r(T-s)}[\sigma^{2}(\pi_{k}^{*}{)}^{2}+n_{k}^{2}\sigma^{2}(\pi_{j}^{*}{)}^{2}-2n_{k}\sigma^{2}\pi_{k}^{*}\pi_{j}^{*}],\;{\underset{―}{d}}_{k1}(s)\;=\;[\eta_{k}q_{k}^{*}-n_{k}\eta_{j}q_{j}^{*}-\beta_{k2}\sigma_{k}^{2}(q_{k}^{*}{)}^{2}e^{r(T-s)}+n_{k}\beta_{j2}\sigma_{j}^{2}(q_{j}^{*}{)}^{2}e^{r(T-s)}]e^{r(T-s)},\;{\underset{―}{d}}_{k2}(s)\;=\;[(\mu -r)(\pi_{k}^{*}-n_{k}\pi_{j}^{*})-\beta_{k1}\sigma^{2}(\pi_{k}^{*}{)}^{2}e^{r(T-s)}+n_{k}\beta_{j1}\sigma^{2}(\pi_{j}^{*}{)}^{2}e^{r(T-s)}]e^{r(T-s)},\;{\overset{―}{d}}_{k1}(s)\;=\;[\eta_{k}q_{k}^{*}-n_{k}\eta_{j}q_{j}^{*}+\beta_{k2}\sigma_{k}^{2}(q_{k}^{*}{)}^{2}e^{r(T-s)}-n_{k}\beta_{j2}\sigma_{j}^{2}(q_{j}^{*}{)}^{2}e^{r(T-s)}]e^{r(T-s)},\;{\overset{―}{d}}_{k2}(s)\;=\;[(\mu -r)(\pi_{k}^{*}-n_{k}\pi_{j}^{*})+\beta_{k1}\sigma^{2}(\pi_{k}^{*}{)}^{2}e^{r(T-s)}-n_{k}\beta_{j1}\sigma^{2}(\pi_{j}^{*}{)}^{2}e^{r(T-s)}]e^{r(T-s)}.$$

Consequently, Eqs (14)–(16) can be readily derived. ◻

**Remark 3.3.** From Eqs (12) and (13), we find that the equilibrium reinsurance strategy $q_{k}^{*}(t)$ is independent of the ambiguity level on the risk asset $\beta_{k1}$, $\beta_{j1}$; while the equilibrium investment strategy $\pi_{k}^{*}(t)$ is independent of the ambiguity level on the insurance risk $\beta_{k2}$, $\beta_{j2}$. And the equilibrium strategy of each insurer $\left(q_{k}^{*}(t),\pi_{k}^{*}(t)\right)$ is independent of the state variable *xk*(*t*) and *xj*(*t*), k,j∈{1,2},k≠j.

**Remark 3.4.** The ambiguity attitudes of the insurers have significant impacts on the equilibrium strategies, the existence of ambiguity-seeking would stimulate the two insurers to choose an aggressive strategy, that is increasing the amount invested in the risky asset and ceding less insurance business risk to the reinsurer. When $\alpha_{k}=1$, i.e., the insurers are extremely ambiguity-averse, the model degenerates to [22], and the optimal reinsurance-investment strategies are in consistent with that in [22].

**Proposition 3.5.** The equilibrium reinsurance strategy $q_{k}^{*}(t)$ is increasing in *nk*, and decreasing in $\beta_{k2}$, $\alpha_{k}$ and $\gamma_{k}$.

*Proof*: We only prove $q_{k}^{*}(t)$ is increasing in *n*_*k*_, and we can similarly prove that $q_{k}^{*}(t)$ and decreasing in $\beta_{k2}$ and $\alpha_{k}$ and $\gamma_{k}$. By differentiating Eq (12) with respect to *n*_*k*_ yields

$$\frac{\partial q_{k}^{*}(t)}{\partial n_{k}}=\frac{\rho \mu_{j}\eta_{j}\sigma_{k}\gamma_{k}\Delta_{1}+n_{j}\rho^{2}\gamma_{k}\gamma_{j}\sigma_{k}^{2}\sigma_{j}e^{r(T-t)}\Delta_{2}}{\Delta_{1}^{2}},$$ (30)

where


$$\Delta_{1}\;=\;\{[\gamma_{k}-({\hat{\alpha }}_{k}-\alpha_{k})\beta_{k2}][\gamma_{j}-({\hat{\alpha }}_{j}-\alpha_{j})\beta_{j2}]-n_{k}n_{j}\rho^{2}\gamma_{k}\gamma_{j}\}\sigma_{k}^{2}\sigma_{j}e^{r(T-t)},$$

$$\Delta_{2}\;=\;\eta_{k}\sigma_{j}[\gamma_{j}-({\hat{\alpha }}_{j}-\alpha_{j})\beta_{j2}]+n_{k}\rho \eta_{j}\sigma_{k}\gamma_{k}.$$

Due to $\frac{1}{2}<\alpha_{k}<1$, we have $\Delta_{1}>0,\Delta_{2}>0$. Then from Eq (30), we obtain $\frac{\partial q_{k}^{*}(t)}{\partial n_{k}}>0$. ◻

**Remark 3.6.** Proposition 3.5 indicates that when the sensitivity coefficient *n*_*k*_ increases, that is the competition between the insurers intensifies, insurers tend to reduce their purchases of reinsurance and take more insurance risks themselves. But in the following three conditions, there is a tendency to increase the purchase of reinsurance and reduce its own risk exposure $q_{k}^{*}(t)$: (1) insurance liabilities are more ambiguous (larger $\beta_{k2}$); (2) insurers are more ambiguity-averse (i.e., larger $\alpha_{k}$); (3) insurers are more risk-averse (i.e., larger $\gamma_{k}$).

Similarly, we can obtain the properties of the equilibrium investment strategy.

**Proposition 3.7.** Equilibrium investment strategy $\pi_{k}^{*}(t)$ about *nk* is monotonically increasing, about $\beta_{k1}$, $\alpha_{k}$ and $\gamma_{k}$ is monotonically decreasing.

**Remark 3.8.** Proposition 3.7 indicates that when the sensitivity coefficient *n*_*k*_ increases, that is the competition between the insurers intensifies, insurers tend to increase the amount invested in risky asset. However, there are three conditions that the insurers tend to reduce the amount invested in the risky asset: (1) risk asset returns are more ambiguous (larger $\beta_{k1}$); (2) insurers are more ambiguous and disgusted (larger $\alpha_{k}$); (3) insurers are more risk averse (larger $\gamma_{k}$).

## 4 Numerical Simulations

In this section, we present several numerical examples to show sensitivity analysis about the equilibrium strategies. Unless otherwise stated, the model parameters are given in S1 Table.

Since the impact of general model parameters for the α-robust equilibrium reinsurance and investment strategies has been studied by [22], in this paper, we focus on the ambiguity parameters $\alpha_{k}$, $\beta_{k1}$, $\beta_{k2}$, the risk coefficient $\gamma_{k}$ and the sensitivity coefficients *n*_*k*_.

S1 Fig displays the different effects of the risk attitude coefficients $\alpha_{k}$ and the sensitivity coefficients *n*_*k*_ on the equilibrium reinsurance strategies of the two insurers at the initial time (t=0), for k∈{1,2}. The results show that the insurer who is more ambiguity-seeking (smaller $\alpha_{k}$) adopt a more aggressive reinsurance strategy by retaining a higher proportion of insurance risk rather than ceding it to the reinsurer. Additionally, the optimal retention level $q_{k}^{*}(0)$ increases as *n*_*k*_ rises, which is consistent with the conclusion in Proposition 3.5. This behavior can be attributed to the fact that insurer with higher sensitivity coefficients *n*_*k*_ is more concerned about outperforming its competitor. As a result, they are willing to take on more risk themselves to widen the wealth gap, rather than spending additional funds to purchase reinsurance protection. In other words, competition drives insurers to become more risk-seeking. Notably, when *n*_*k*_ = 0, indicating no concern for relative performance, the insurer tends to purchase the maximum amount of reinsurance to minimize their own risk exposure.

S2 Fig illustrates the influence of the ambiguity aversion coefficients $\beta_{k2}$ and the risk aversion coefficients $\gamma_{k}$ on the equilibrium reinsurance strategy of two insurers at the initial time, for k∈{1,2}. It is evident that, for a given level of ambiguity aversion, the optimal retention proportion $q_{k}^{*}(0)$ decreases as the risk aversion coefficient $\gamma_{k}$ increases. This trend can be attributed to the fact that an insurer with a higher risk aversion coefficient $\gamma_{k}$ prefers to bear less insurance risk and thus cedes more risk to the reinsurer. Moreover, the optimal retention proportion $q_{k}^{*}(0)$ also decreases with increasing ambiguity aversion parameter $\beta_{k2}$. This finding aligns with our intuition, as insurer with higher levels of ambiguity aversion is more inclined to purchase additional reinsurance to mitigate the adverse impacts of potential model misspecification. Particularly, $\beta_{k2}=0$, for k∈{1,2}, corresponds to an ambiguity-neutral insurer who cedes the most insurance risk to the reinsurer.

S3 Fig displays the impacts of the ambiguity attitude coefficients $\alpha_{k}$ and the sensitivity coefficients *n*_*k*_ on the equilibrium investment strategy $\pi_{k}^{*}(0)$, for k∈{1,2}. Firstly, it is observed that the equilibrium investment strategy $\pi_{k}^{*}(0)$ is a decreasing function of $\alpha_{k}$ when *n*_*k*_ is held constant. A larger $\alpha_{k}$ indicates a higher degree of ambiguity aversion on the part of the insurer, leading to a reduction in the amount invested in the risky asset as a means of avoiding uncertainty. Moreover, when *n*_*k*_ is fixed, the equilibrium investment strategy $\pi_{k}^{*}(0)$ is seen to decrease as $\alpha_{k}$ grows. The larger $\alpha_{k}$ is, the more ambiguous averse the insurer *k* is. Therefore, he tends to reduce the amount invested in the risky asset to avoid uncertainty. Additionally, for a given $\alpha_{k}$, the equilibrium investment strategy $\pi_{k}^{*}(0)$ is an increasing function of *n*_*k*_. This is attributed to the competitive environment, which encourages insurers to be more risk-seeking. Insurer with a higher sensitivity coefficient *n*_*k*_ is inclined to increase its investment in the risky asset to enhance its prospects of outperforming its competitor at the terminal day. These findings are consistent with Proposition 3.7.

S4 Fig shows that the effects of parameters $\beta_{k1}$ (i.e., the ambiguity aversion coefficients) and parameters $\gamma_{k}$ (i.e., risk aversion coefficients) on the equilibrium investment strategy $\pi_{k}^{*}(0)$, for k∈{1,2}. As is shown in S4 Fig, for a fixed ambiguity aversion coefficient, $\pi_{k}^{*}(0)$ will decrease with the increase of $\gamma_{k}$. This is because the insurer with a larger risk-averse coefficient $\gamma_{k}$ which means it is more risk-averse tends to reduce the money invested in the risky asset. Moreover, $\pi_{k}^{*}(0)$ decreases as the ambiguity aversion parameter $\beta_{k2}$ increases, because the insurer with higher levels of ambiguity aversion is prone to decrease the money invested in the risky asset to offset the adverse effects of model misspecification.

## 5 Conclusion

In this paper, we study the α-robust non-zero-sum reinsurance and investment game involving two competing insurers, both of them adopt the α-maxmin mean-variance utility. We formulate the optimization problem, and by using techniques in stochastic control theory, we derive the extended HJB equations, and obtain the closed-form solutions of the optimal reinsurance-investment strategies and value functions. The numerical results reveal several insightful findings. The results show that the optimal reinsurance and investment strategies are directly proportional to the sensitivity coefficient of competition, while they are inversely proportional to the risk attitude coefficients, the ambiguity aversion coefficients and the risk aversion coefficient. The competition makes insurers more risk-seeking, that is, the insurer who is more concerned about the relative performance and aim to outperform its competitor would adopt a more aggressive strategy, Specifically, this insurer would retain more insurance risk and invest more wealth in the risky asset. And the insurer with a greater inclination towards ambiguity would also adopt a more aggressive strategy.

Several extensions of this paper can be explored in the future research, such as the stock price process obeying other models and bounded memory, etc. We leave these extensions for future work.

## Supporting information

S1 Appendix Proof of Theorem 3.1 (PDF)

S1 Table Values of basic parameters. (PDF)

S1 FigEffects of the risk attitude coefficients $\alpha_{k}$ and the sensitivity coefficients $n_{k}$ on the optimal reinsurance strategy of insurer *k*, for k∈{1,2}.(TIF)

S2 FigEffects of the ambiguity aversion coefficients $\beta_{k2}$ and risk aversion coefficients $\gamma_{k}$ on the optimal reinsurance strategy of insurer *k*, for k∈{1,2}.(TIF)

S3 FigEffects of the ambiguity attitude coefficients $\alpha_{k}$ and the sensitivity coefficients $n_{k}$ on the optimal investment strategy of insurer *k*, for k∈{1,2}.(TIF)

S4 FigEffects of the ambiguity aversion coefficients $\beta_{k2}$ and risk aversion coefficients $\gamma_{k}$ on the optimal reinsurance strategy of insurer *k*, for k∈{1,2}.(TIF)
