# Supplementary material for: Reinsurance–investment game between two α-maxmin mean–variance insurers
Source: PLoS One. 2025 Jun 27;20(6):e0326125. doi: 10.1371/journal.pone.0326125 (PMC12204578; doi:10.1371/journal.pone.0326125)
Supplement: S1 Appendix — (PDF) [file pone.0326125.s001.pdf]

S1 Appendix. The proof of Theorem 3.1.

Theorem 3.1. (*Verification Theorem*) For Problem 2.1, if there are real-valued functions $V_k(t,x_k), \underline{g}_k(t,x_k), \overline{g}_k(t,x_k) \in C^{1,2}([0,T]\times\mathbb{R})$ satisfy the following conditions:

(1) For any $(t,x_k)\in[0,T]\times\mathbb{R}$,

$$\begin{aligned}\sup_{u_k\in\mathcal{U}_k}\Bigg\{\alpha_k \inf_{\phi_k\in\Phi_k}\Big[&\mathcal{L}^{u_k,u_j^*,\phi_k,\underline{\phi}_j^*}V_k(t,x_k)-\frac{\gamma_k}{2}\mathcal{L}^{u_k,u_j^*,\phi_k,\underline{\phi}_j^*}\underline{g}_k^2(t,x_k)\\ &+\gamma_k\underline{g}_k(t,x_k)\mathcal{L}^{u_k,u_j^*,\phi_k,\underline{\phi}_j^*}\underline{g}_k(t,x_k)+h_\beta(\phi_k)\Big]\\ +\widehat{\alpha}_k\sup_{\phi_k\in\Phi_k}\Big[&\mathcal{L}^{u_k,u_j^*,\phi_k,\overline{\phi}_j^*}V_k(t,x_k)-\frac{\gamma_k}{2}\mathcal{L}^{u_k,u_j^*,\phi_k,\overline{\phi}_j^*}\overline{g}_k^2(t,x_k)\\ &+\gamma_k\underline{g}_k(t,x_k)\mathcal{L}^{u_k,u_j^*,\phi_k,\overline{\phi}_j^*}\overline{g}_k(t,x_k)-h_\beta(\phi_k)\Big]\Bigg\}=0,\end{aligned}\tag{10}$$

(2) For any $(t,x_k)\in[0,T]\times\mathbb{R}$,

$$\begin{cases}V_k(T,x_k)=x_k,\\ \mathcal{L}^{u_k^*,u_j^*,\underline{\phi}_k^*,\underline{\phi}_j^*}\underline{g}_k(t,x_k)=\mathcal{L}^{u_k^*,u_j^*,\overline{\phi}_k^*,\overline{\phi}_j^*}\overline{g}_k(t,x_k)=0,\\ \underline{g}_k(T,x_k)=\overline{g}_k(T,x_k)=x_k.\end{cases}\tag{11}$$

(3) For any $(t,x_k)\in[0,T]\times\mathbb{R}$, $u_k^*(t)$, $\underline{\phi}_k^*(t)$, $\overline{\phi}_k^*(t)$, $\mathcal{L}^{u_k^*,u_j^*,\underline{\phi}_k^*,\underline{\phi}_j^*}V(t,x_k)$, $\mathcal{L}^{u_k^*,u_j^*,\overline{\phi}_k^*,\overline{\phi}_j^*}V(t,x_k)$, $\mathcal{L}^{u_k^*,u_j^*,\underline{\phi}_k^*,\underline{\phi}_j^*}\underline{g}_k^2(t,x_k)$ and $\mathcal{L}^{u_k^*,u_j^*,\overline{\phi}_k^*,\overline{\phi}_j^*}\overline{g}_k^2(t,x_k)$ are deterministic functions of t and independent of x_k.

(4)$\underline{\phi}_k^*=\underline{\phi}_k^{u_k^*}$ and $\overline{\phi}_k^*=\overline{\phi}_k^{u_k^*}$.

Then u_k^* is the α-robust equilibrium strategy, $V_k(t,x_k)=J_k^{u_k^*,u_j^*}(t,x_k)$ is the equilibrium value function of insurer k. Besides, $\underline{g}_k(t,x_k)=\mathbb{E}_{t,x_k}^{\underline{\phi}_k^*}\big[X_k^{u_k^*,u_j^*}(T)\big]$, $\overline{g}_k(t,x_k)=\mathbb{E}_{t,x_k}^{\overline{\phi}_k^*}\big[X_k^{u_k^*,u_j^*}(T)\big]$.

Proof. The proof of Theorem 3.1 is divided into two steps. First, we show that $\underline{g}_k(t,x_k)=\mathbb{E}_{t,x_k}^{\underline{\phi}_k^*}\big[X_k^{u_k^*,u_j^*}(T)\big]$, $\overline{g}_k(t,x_k)=\mathbb{E}_{t,x_k}^{\overline{\phi}_k^*}\big[X_k^{u_k^*,u_j^*}(T)\big]$, and $V_k(t,x_k)=J_k^{u_k^*,u_j^*}(t,x_k)$. Second, we prove that u_k^* is the equilibrium reinsurance–investment strategy of insurer k.

According to Dynkin's formula and the second relation of Eqs 11, we have

$$\begin{aligned}&\mathbb{E}_{t,x_k}^{\underline{\phi}_k^*}\left[\underline{g}_k\left(T,X_k^{u_k^*,u_j^*}(T)\right)\right]\\ =&\underline{g}_k(t,x_k)+\mathbb{E}_{t,x_k}^{\underline{\phi}_k^*}\left[\int_t^T\mathcal{L}_k^{u_k^*,u_j^*,\underline{\phi}_k^*,\underline{\phi}_j^*}\underline{g}_k(s,X^{u_k^*,u_j^*}(s))\,ds\right]\\ =&\underline{g}_k(t,x_k).\end{aligned}\tag{A.1}$$

Taking into account the third relation of Eqs 11, we obtain

$$\underline{g}_k(t,x_k)=\mathbb{E}_{t,x_k}^{\underline{\phi}_k^*}\left[\underline{g}_k\left(T,X_k^{u_k^*,u_j^*}(T)\right)\right]=\mathbb{E}_{t,x_k}^{\underline{\phi}_k^*}\left[X_k^{u_k^*,u_j^*}(T)\right].\tag{A.2}$$

We can similarly prove that $\overline{g}_k(t,x_k) = \mathbb{E}_{t,x_k}^{\overline{\phi}_k^*}\big[X_k^{u_k^*,u_j^*}(T)\big]$.

Noting that $(u_k^*, \underline{\phi}_k^*, \overline{\phi}_k^*)$ solves the optimization problem in the left-hand side of Eq 10 and the second relation of Eqs 11, we can obtain

$$\begin{aligned} &\alpha_k\Big\{\mathcal{L}^{u_k^*,u_j^*,\underline{\phi}_k^*,\underline{\phi}_j^*}V_k(t,x_k) - \frac{\gamma_k}{2}\mathcal{L}^{u_k^*,u_j^*,\underline{\phi}_k^*,\underline{\phi}_j^*}\underline{g}_k^2(t,x_k) + h_\beta(\underline{\phi}_k^*)\Big\} \\ &+\widehat{\alpha}_k\Big\{\mathcal{L}^{u_k^*,u_j^*,\overline{\phi}_k^*,\overline{\phi}_j^*}V_k(t,x_k) - \frac{\gamma_k}{2}\mathcal{L}^{u_k^*,u_j^*,\overline{\phi}_k^*,\overline{\phi}_j^*}\overline{g}_k^2(t,x_k) - h_\beta(\overline{\phi}_k^*)\Big\} = 0. \end{aligned} \tag{A.3}$$

By the condition $V_k(T,x_k) = x_k$, and using Dynkin's formula again, we have

$$\begin{aligned} \mathbb{E}_{t,x_k}^{\underline{\phi}_k^*}\left[V_k\left(T, X_k^{u_k^*,u_j^*}(T)\right)\right] &= V_k(t,x_k) + \mathbb{E}_{t,x_k}^{\underline{\phi}_k^*}\left[\int_t^T \mathcal{L}_k^{u_k^*,u_j^*,\underline{\phi}_k^*,\underline{\phi}_j^*}V_k(s, X^{u_k^*,u_j^*}(s))\,ds\right] \\ &= \mathbb{E}_{t,x_k}^{\underline{\phi}_k^*}\left[X_k^{u_k^*,u_j^*}(T)\right], \\ \mathbb{E}_{t,x_k}^{\overline{\phi}_k^*}\left[V_k\left(T, X_k^{u_k^*,u_j^*}(T)\right)\right] &= V_k(t,x_k) + \mathbb{E}_{t,x_k}^{\overline{\phi}_k^*}\left[\int_t^T \mathcal{L}_k^{u_k^*,u_j^*,\overline{\phi}_k^*,\overline{\phi}_j^*}V_k(s, X^{u_k^*,u_j^*}(s))\,ds\right] \\ &= \mathbb{E}_{t,x_k}^{\underline{\phi}_k^*}\left[X_k^{u_k^*,u_j^*}(T)\right]. \end{aligned}$$

A linear combination of the above two equations yields

$$\begin{aligned} &\alpha_k\mathbb{E}_{t,x_k}^{\underline{\phi}_k^*}\left[X_k^{u_k^*,u_j^*}(T)\right] + \widehat{\alpha}_k\mathbb{E}_{t,x_k}^{\overline{\phi}_k^*}\left[X_k^{u_k^*,u_j^*}(T)\right] \\ =&V_k(t,x_k) + \alpha_k\mathbb{E}_{t,x_k}^{\underline{\phi}_k^*}\left[\int_t^T \mathcal{L}_k^{u_k^*,u_j^*,\underline{\phi}_k^*,\underline{\phi}_j^*}V_k(s, X^{u_k^*,u_j^*}(s))\,ds\right] \\ &+ \widehat{\alpha}_k\mathbb{E}_{t,x_k}^{\overline{\phi}_k^*}\left[\int_t^T \mathcal{L}_k^{u_k^*,u_j^*,\overline{\phi}_k^*,\overline{\phi}_j^*}V_k(s, X^{u_k^*,u_j^*}(s))\,ds\right]. \end{aligned}$$

Now plugging Eq (A.3) into the last equation, we can obtain

$$\begin{aligned} &V_k(t,x_k) \\ =&\alpha_k\Big\{\mathbb{E}_{t,x_k}^{\underline{\phi}_k^*}\left[X_k^{u_k^*,u_j^*}(T)\right] - \frac{\gamma_k}{2}\mathbb{E}_{t,x_k}^{\underline{\phi}_k^*}\left[\int_t^T \mathcal{L}^{u_k^*,u_j^*,\underline{\phi}_k^*,\underline{\phi}_j^*}\underline{g}_k^2(s, X^{u_k^*,u_j^*}(s))ds\right] + \int_t^T h_\beta(\underline{\phi}_k^*(s))\,ds\Big\} \\ &+\widehat{\alpha}_k\Big\{\mathbb{E}_{t,x_k}^{\overline{\phi}_k^*}\left[X_k^{u_k^*,u_j^*}(T)\right] - \frac{\gamma_k}{2}\mathbb{E}_{t,x_k}^{\overline{\phi}_k^*}\left[\int_t^T \mathcal{L}^{u_k^*,u_j^*,\overline{\phi}_k^*,\overline{\phi}_j^*}\overline{g}_k^2(s, X^{u_k^*,u_j^*}(s))ds\right] - \int_t^T h_\beta(\overline{\phi}_k^*(s))\,ds\Big\} \end{aligned} \tag{A.4}$$

Moreover, by the the third relation of Eqs 11, Dynkin's formula and Eq (A.2), we have

$$\begin{aligned} &\alpha_k\mathbb{E}_{t,x_k}^{\underline{\phi}_k^*}\left[\left(X_k^{u_k^*,u_j^*}(T)\right)^2\right] + \widehat{\alpha}_k\mathbb{E}_{t,x_k}^{\overline{\phi}_k^*}\left[\left(X_k^{u_k^*,u_j^*}(T)\right)^2\right] \\ =&\alpha_k\mathbb{E}_{t,x_k}^{\underline{\phi}_k^*}\left[\underline{g}_k^2\left(X_k^{u_k^*,u_j^*}(T)\right)\right] + \widehat{\alpha}_k\mathbb{E}_{t,x_k}^{\overline{\phi}_k^*}\left[\overline{g}_k^2\left(X_k^{u_k^*,u_j^*}(T)\right)\right] \\ =&\alpha_k\underline{g}_k^2(t,x_k) + \alpha_k\mathbb{E}_{t,x_k}^{\underline{\phi}_k^*}\left[\int_t^T \mathcal{L}^{u_k^*,u_j^*,\underline{\phi}_k^*,\underline{\phi}_j^*}\underline{g}_k^2(s, X^{u_k^*,u_j^*}(s))ds\right] \\ &+ \widehat{\alpha}_k\overline{g}_k^2(t,x_k) + \alpha_k\mathbb{E}_{t,x_k}^{\overline{\phi}_k^*}\left[\int_t^T \mathcal{L}^{u_k^*,u_j^*,\overline{\phi}_k^*,\underline{\phi}_j^*}\overline{g}_k^2(s, X^{u_k^*,u_j^*}(s))ds\right] \\ =&\alpha_k\left(\mathbb{E}_{t,x_k}^{\underline{\phi}_k^*}\left[X_k^{u_k^*,u_j^*}(T)\right]\right)^2 + \alpha_k\mathbb{E}_{t,x_k}^{\underline{\phi}_k^*}\left[\int_t^T \mathcal{L}^{u_k^*,u_j^*,\underline{\phi}_k^*,\underline{\phi}_j^*}\underline{g}_k^2(s, X^{u_k^*,u_j^*}(s))ds\right] \\ &+ \widehat{\alpha}_k\left(\mathbb{E}_{t,x_k}^{\overline{\phi}_k^*}\left[X_k^{u_k^*,u_j^*}(T)\right]\right)^2 + \alpha_k\mathbb{E}_{t,x_k}^{\overline{\phi}_k^*}\left[\int_t^T \mathcal{L}^{u_k^*,u_j^*,\underline{\phi}_k^*,\overline{\phi}_j^*}\overline{g}_k^2(s, X^{u_k^*,u_j^*}(s))ds\right]. \end{aligned} \tag{A.5}$$

Note that Eq (A.5) is equivalent to

$$
\begin{aligned}
&\alpha_k \mathrm{Var}^{\underline{\phi}^*_k}_{t,x_k}\left[X_k^{u_k^*,u_j^*}(T)\right] + \widehat{\alpha}_k \mathrm{Var}^{\overline{\phi}^*_k}_{t,x_k}\left[X_k^{u_k^*,u_j^*}(T)\right] \\
=&\alpha_k \mathbb{E}^{\underline{\phi}^*_k}_{t,x_k}\left[\int_t^T \mathcal{L}^{u_k^*,u_j^*,\underline{\phi}^*_k,\underline{\phi}^*_j}\underline{g}_k^2(s, X^{u_k^*,u_j^*}(s))ds\right] \\
&+ \alpha_k \mathbb{E}^{\overline{\phi}^*_k}_{t,x_k}\left[\int_t^T \mathcal{L}^{u_k^*,u_j^*,\underline{\phi}^*_k,\overline{\phi}^*_j}\overline{g}_k^2(s, X^{u_k^*,u_j^*}(s))ds\right].
\end{aligned}
\tag{A.6}
$$

Finally, putting Eq (A.6) into Eq (A.4) yields

$$
\begin{aligned}
&V_k(t,x_k) \\
=&\alpha_k\left\{\mathbb{E}^{\underline{\phi}^*_k}_{t,x_k}\left[X_k^{u_k^*,u_j^*}(T)\right] - \frac{\gamma_k}{2}\mathrm{Var}^{\underline{\phi}^*_k}_{t,x_k}\left[X_k^{u_k^*,u_j^*}(T)\right] + \int_t^T h_\beta(\underline{\phi}^*_k(s))\,ds\right\} \\
&+ \widehat{\alpha}_k\left\{\mathbb{E}^{\overline{\phi}^*_k}_{t,x_k}\left[X_k^{u_k^*,u_j^*}(T)\right] - \frac{\gamma_k}{2}\mathrm{Var}^{\overline{\phi}^*_k}_{t,x_k}\left[X_k^{u_k^*,u_j^*}(T)\right] - \int_t^T h_\beta(\overline{\phi}^*_k(s))\,ds\right\} \\
=&J_k^{u_k^*,u_j^*}(t,x_k).
\end{aligned}
\tag{A.7}
$$

Second, we are going to prove that u_I^* is an equilibrium reinsurance-investment strategy. Before that, we state some properties as following. (1) The function $\overline{J}_k^{u_k,\overline{\phi}_k,u_j,\overline{\phi}_j}(t,x_k) - \underline{J}_k^{u_k,\underline{\phi}_k,u_j,\underline{\phi}_j}(t,x_k)$ is independent of x_k. (2) Consider the deterministic reinsurance–investment strategy

$$
u_k^\epsilon(v) := \begin{cases} \hat{u}_k(v), & v \in [t, t+\epsilon), \\ u_k^*(v), & v \in [t+\epsilon, T], \end{cases}
$$

where $\hat{u}_k \in \mathcal{U}_k$. Similar to the Lemma A.2 in [1], it can be proved that the density generator functions attaining the infimum and supremum in Eq (9) are given respectively by

$$
\underline{\phi}_k^{u_k^\epsilon}(v) := \begin{cases} \underline{\phi}_k^{\hat{u}_k}(v), & v \in [t, t+\epsilon), \\ \underline{\phi}_k^{u_k^*}(v), & v \in [t+\epsilon, T], \end{cases}
\tag{A.8}
$$

and

$$
\overline{\phi}_k^{u_k^\epsilon}(v) := \begin{cases} \overline{\phi}_k^{\hat{u}_k}(v), & v \in [t, t+\epsilon), \\ \overline{\phi}_k^{u_k^*}(v), & v \in [t+\epsilon, T], \end{cases}
\tag{A.9}
$$

and for any measurable function $f_k : \mathbb{R} \to \mathbb{R}$, we have

$$
\mathbb{E}^{\underline{\phi}_k^{u_k^\epsilon}}_{t,x_k}\left[f\left(X_k^{u_k^\epsilon,u_j^*}(T)\right)\right] = \mathbb{E}^{\underline{\phi}_k^{\hat{u}_k}}_{t,x_k}\left[\mathbb{E}^{\underline{\phi}^*_k}_{t+\epsilon, X_k^{\hat{u}_k}(t+\epsilon)}\left[f\left(X_k^{u_k^*,u_j^*}(T)\right)\right]\right],
\tag{A.10}
$$

and

$$
\mathbb{E}^{\overline{\phi}_k^{u_k^\epsilon}}_{t,x_k}\left[f\left(X_k^{u_k^\epsilon,u_j^*}(T)\right)\right] = \mathbb{E}^{\overline{\phi}_k^{\hat{u}_k}}_{t,x_k}\left[\mathbb{E}^{\overline{\phi}^*_k}_{t+\epsilon, X_k^{\hat{u}_k}(t+\epsilon)}\left[f\left(X_k^{u_k^*,u_j^*}(T)\right)\right]\right].
\tag{A.11}
$$

Here, we omit the proofs of these two conclusions. The interested readers can refer to Appendix A in [1].

In order to prove that u_k^* is an equilibrium strategy, according to Definition 2.3, it suffices to show that

$$J_k^{u_k^\epsilon,u_j^*}(t,x_k) - J_k^{u_k^*,u_j^*}(t,x_k) \le o(\epsilon).$$

To this end, we first derive the expression of $J_k^{u_k^\epsilon,u_j^*}(t,x_k) - J_k^{u_k^*,u_j^*}(t,x_k)$. By Eq (9), we have

$$\begin{aligned}
&\underline{J}_k^{u_k^\epsilon,u_j^*,\underline{\phi}_k^{u_k^\epsilon},\underline{\phi}_j^*}(t,x_k)\\
=&\mathbb{E}_{t,x_k}^{\underline{\phi}_k^{u_k^\epsilon},\underline{\phi}_j^*}\left[X_k^{u_k^\epsilon,u_j^*}(T) - \frac{\gamma_k}{2}\left(X_k^{u_k^\epsilon,u_j^*}(T)\right)^2\right] + \frac{\gamma_k}{2}\left(\mathbb{E}_{t,x_k}^{\underline{\phi}_k^{u_k^\epsilon},\underline{\phi}_j^*}\left[X_k^{u_k^\epsilon,u_j^*}(T)\right]\right)^2 + \int_t^T h_\beta(\underline{\phi}_k^{u_k^\epsilon,u_j^*}(s))\,ds\\
=&\mathbb{E}_{t,x_k}^{\underline{\phi}_k^{\hat{u}_k},\underline{\phi}_j^*}\left[\mathbb{E}_{t+\epsilon,X_k^{\hat{u}_k}(t+\epsilon)}^{\underline{\phi}_k^*,\underline{\phi}_j^*}\left[X_k^{u_k^*,u_j^*}(T) - \frac{\gamma_k}{2}\left(X_k^{u_k^*,u_j^*}(T)\right)^2\right]\right]\\
&+\frac{\gamma_k}{2}\left(\mathbb{E}_{t,x_k}^{\underline{\phi}_k^{\hat{u}_k},\underline{\phi}_j^*}\left[\mathbb{E}_{t+\epsilon,X_k^{\hat{u}_k}(t+\epsilon)}^{\underline{\phi}_k^*,\underline{\phi}_j^*}\left[X_k^{u_k^*,u_j^*}(T)\right]\right]\right)^2\\
&+\int_t^{t+\epsilon} h_\beta(\underline{\phi}_k^{\hat{u}_k}(s))\,ds + \int_{t+\epsilon}^T h_\beta(\underline{\phi}_k^{u_k^*}(s))\,ds\\
=&\mathbb{E}_{t,x_k}^{\underline{\phi}_k^{\hat{u}_k},\underline{\phi}_j^*}\left[\underline{J}_k^{u_k^*,u_j^*,\underline{\phi}_k^*,\underline{\phi}_j^*}(t+\epsilon,X_k^{\hat{u}_k}(t+\epsilon))\right] - \frac{\gamma_k}{2}\mathbb{E}_{t,x_k}^{\underline{\phi}_k^{\hat{u}_k},\underline{\phi}_j^*}\left[\left(\mathbb{E}_{t+\epsilon,X_k^{\hat{u}_k}(t+\epsilon)}^{\underline{\phi}_k^*,\underline{\phi}_j^*}\left[X_k^{u_k^*,u_j^*}(T)\right)^2\right]\right]\\
&+\frac{\gamma_k}{2}\left(\mathbb{E}_{t,x_k}^{\underline{\phi}_k^{\hat{u}_k},\underline{\phi}_j^*}\left[\mathbb{E}_{t+\epsilon,X_k^{\hat{u}_k}(t+\epsilon)}^{\underline{\phi}_k^*,\underline{\phi}_j^*}\left[X_k^{u_k^*,u_j^*}(T)\right]\right]\right)^2 + \int_t^{t+\epsilon} h_\beta(\underline{\phi}_k^{\hat{u}_k}(s))\,ds
\end{aligned}$$

$$\begin{aligned}
=&\mathbb{E}_{t,x_k}^{\underline{\phi}_k^{\hat{u}_k},\underline{\phi}_j^*}\left[\underline{J}_k^{u_k^*,u_j^*,\underline{\phi}_k^*,\underline{\phi}_j^*}(t+\epsilon,X_k^{\hat{u}_k}(t+\epsilon))\right] - \frac{\gamma_k}{2}\mathbb{E}_{t,x_k}^{\underline{\phi}_k^{\hat{u}_k},\underline{\phi}_j^*}\left[\underline{g}_k^2(t+\epsilon,X_k^{\hat{u}_k}(t+\epsilon))\right]\\
&+\frac{\gamma_k}{2}\left(\mathbb{E}_{t,x_k}^{\underline{\phi}_k^{\hat{u}_k},\underline{\phi}_j^*}\left[\underline{g}_k\big(t+\epsilon,X_k^{\hat{u}_k}(t+\epsilon)\big)\right]\right)^2 + \int_t^{t+\epsilon} h_\beta(\underline{\phi}_k^{\hat{u}_k}(s))\,ds,
\end{aligned}$$

where the last equality is obtained by using Eq (A.1). The same discussion leads to

$$\begin{aligned}
&\overline{J}_k^{u_k^\epsilon,u_j^*,\overline{\phi}_k^{u_k^\epsilon},\overline{\phi}_j^*}(t,x_k)\\
=&\mathbb{E}_{t,x_k}^{\overline{\phi}_k^{\hat{u}_k},\overline{\phi}_j^*}\left[\overline{J}_k^{u_k^*,u_j^*,\overline{\phi}_k^*,\overline{\phi}_j^*}(t+\epsilon,X_k^{\hat{u}_k}(t+\epsilon))\right] - \frac{\gamma_k}{2}\mathbb{E}_{t,x_k}^{\phi_k^{\hat{u}_k},\overline{\phi}_j^*}\left[\overline{g}_k^2(t+\epsilon,X_k^{\hat{u}_k}(t+\epsilon))\right]\\
&+\frac{\gamma_k}{2}\left(\mathbb{E}_{t,x_k}^{\overline{\phi}_k^{\hat{u}_k},\overline{\phi}_j^*}\left[\overline{g}_k\big(t+\epsilon,X_k^{\hat{u}_k}(t+\epsilon)\big)\right]\right)^2 - \int_t^{t+\epsilon} h_\beta(\overline{\phi}_k^{\hat{u}_k}(s))\,ds.
\end{aligned}\tag{A.12}$$

A linear combination of the above two equations yields

$$\begin{aligned}
&J_k^{u_k^\epsilon,u_j^*}(t,x_k) = \alpha\underline{J}_k^{u_k^\epsilon,u_j^*,\underline{\phi}_k^{u_k^\epsilon},\underline{\phi}_j^*}(t,x_k) + \widehat{\alpha}\overline{J}_k^{u_k^\epsilon,u_j^*,\overline{\phi}_k^{u_k^\epsilon},\overline{\phi}_j^*}(t,x_k)\\
=&\alpha\mathbb{E}_{t,x_k}^{\underline{\phi}_k^{\hat{u}_k},\underline{\phi}_j^*}\left[\underline{J}_k^{u_k^*,u_j^*,\underline{\phi}_k^*,\underline{\phi}_j^*}(t+\epsilon,X_k^{\hat{u}_k}(t+\epsilon))\right] + \widehat{\alpha}\mathbb{E}_{t,x_k}^{\overline{\phi}_k^{\hat{u}_k},\overline{\phi}_j^*}\left[\overline{J}_k^{u_k^*,u_j^*,\overline{\phi}_k^*,\overline{\phi}_j^*}(t+\epsilon,X_k^{\hat{u}_k}(t+\epsilon))\right]\\
&-\frac{\alpha\gamma_k}{2}\mathbb{E}_{t,x_k}^{\underline{\phi}_k^{\hat{u}_k},\underline{\phi}_j^*}\left[\underline{g}_k^2(t+\epsilon,X_k^{\hat{u}_k}(t+\epsilon))\right] + \frac{\alpha\gamma_k}{2}\left(\mathbb{E}_{t,x_k}^{\underline{\phi}_k^{\hat{u}_k},\underline{\phi}_j^*}\left[\underline{g}_k\big(t+\epsilon,X_k^{\hat{u}_k}(t+\epsilon)\big)\right]\right)^2\\
&-\frac{\widehat{\alpha}\gamma_k}{2}\mathbb{E}_{t,x_k}^{\phi_k^{\hat{u}_k},\overline{\phi}_j^*}\left[\overline{g}_k^2(t+\epsilon,X_k^{\hat{u}_k}(t+\epsilon))\right] + \frac{\widehat{\alpha}\gamma_k}{2}\left(\mathbb{E}_{t,x_k}^{\overline{\phi}_k^{\hat{u}_k},\overline{\phi}_j^*}\left[\overline{g}_k\big(t+\epsilon,X_k^{\hat{u}_k}(t+\epsilon)\big)\right]\right)^2\\
&+\alpha\int_t^{t+\epsilon} h_\beta(\underline{\phi}_k^{\hat{u}_k}(s))\,ds - \widehat{\alpha}\int_t^{t+\epsilon} h_\beta(\overline{\phi}_k^{\hat{u}_k}(s))\,ds,
\end{aligned}$$

Then we have

$$J_k^{u_k^\epsilon,u_j^*}(t,x_k) - J_k^{u_k^*,u_j^*}(t,x_k) = J_k^{u_k^\epsilon,u_j^*}(t,x_k) - \alpha \underline{J}_k^{u_k^*,u_j^*,\underline{\phi}_k^*,\underline{\phi}_j^*}(t,x_k) - \widehat{\alpha}\overline{J}_k^{u_k^*,u_j^*,\overline{\phi}_k^*,\overline{\phi}_j^*}(t,x_k)$$
$$:= N_\epsilon,$$

where

$$\begin{aligned}
N_\epsilon := & \alpha \left\{ \mathbb{E}_{t,x_k}^{\underline{\phi}_k^{\hat{u}_k},\underline{\phi}_j^*} \left[\underline{J}_k^{u_k^*,u_j^*,\underline{\phi}_k^*,\underline{\phi}_j^*}(t+\epsilon, X_k^{\hat{u}_k}(t+\epsilon)) \right] - \underline{J}_k^{u_k^*,u_j^*,\underline{\phi}_k^*,\underline{\phi}_j^*}(t,x_k) \right\} \\
& + \widehat{\alpha} \left\{ \mathbb{E}_{t,x_k}^{\overline{\phi}_k^{\hat{u}_k},\overline{\phi}_j^*} \left[\overline{J}_k^{u_k^*,u_j^*,\overline{\phi}_k^*,\overline{\phi}_j^*}(t+\epsilon, X_k^{\hat{u}_k}(t+\epsilon)) \right] - \overline{J}_k^{u_k^*,u_j^*,\overline{\phi}_k^*,\overline{\phi}_j^*}(t,x_k) \right\} \\
& - \frac{\alpha\gamma_k}{2} \left\{ \mathbb{E}_{t,x_k}^{\underline{\phi}_k^{\hat{u}_k},\underline{\phi}_j^*} \left[\underline{g}_k^2(t+\epsilon, X_k^{\hat{u}_k}(t+\epsilon)) \right] - \underline{g}_k^2(t,x_k) \right\} \\
& + \frac{\alpha\gamma_k}{2} \left\{ \left(\mathbb{E}_{t,x_k}^{\underline{\phi}_k^{\hat{u}_k},\underline{\phi}_j^*} \left[\underline{g}_k\big(t+\epsilon, X_k^{\hat{u}_k}(t+\epsilon)\big) \right] \right)^2 - \underline{g}_k^2(t,x_k) \right\}
\end{aligned}$$

$$\begin{aligned}
& - \frac{\widehat{\alpha}\gamma_k}{2} \left\{ \mathbb{E}_{t,x_k}^{\underline{\phi}_k^{\hat{u}_k},\overline{\phi}_j^*} \left[\overline{g}_k^2(t+\epsilon, X_k^{\hat{u}_k}(t+\epsilon)) \right] - \overline{g}_k^2(t,x_k) \right\} \\
& + \frac{\widehat{\alpha}\gamma_k}{2} \left\{ \left(\mathbb{E}_{t,x_k}^{\overline{\phi}_k^{\hat{u}_k},\overline{\phi}_j^*} \left[\overline{g}_k\big(t+\epsilon, X_k^{\hat{u}_k}(t+\epsilon)\big) \right] \right)^2 - \overline{g}_k^2(t,x_k) \right\} \\
& + \alpha \int_t^{t+\epsilon} h_\beta(\underline{\phi}_k^{\hat{u}_k}(s))\, ds - \widehat{\alpha} \int_t^{t+\epsilon} h_\beta(\overline{\phi}_k^{\hat{u}_k}(s))\, ds
\end{aligned} \tag{A.13}$$

For the ease of notation, $\forall \psi_k(t,x_k) \in \mathcal{C}^{1,2}([0,T]\times\mathbb{R})$, we define an operator

$$\mathcal{L}_\epsilon^{u_k,u_j,\phi_k,\phi_j}\psi_k(t,x_k) := \mathbb{E}_{t,x_k}^{\phi_k,\phi_j}\left[\psi_k\big(t+\epsilon, X_k^{u_k,u_j}(t+\epsilon)\big)\right] - \psi_k(t,x_k),$$

where $u_k \in \mathcal{U}_k$, $\phi_k \in \Phi_k$, and $\epsilon > 0$.

By the results in Eq (A.8) and Eq (A.9), we have

$$\begin{aligned}
\mathcal{L}_\epsilon^{\widehat{u}_k,u_j,\underline{\phi}_k^{u_k^\epsilon},\phi_j}\psi_k(t,x_k) &= \mathcal{L}_\epsilon^{\widehat{u}_k,u_j,\underline{\phi}_k^{\widehat{u}_k},\phi_j}\psi_k(t,x_k), \\
\mathcal{L}_\epsilon^{\widehat{u}_k,u_j,\overline{\phi}_k^{u_k^\epsilon},\phi_j}\psi_k(t,x_k) &= \mathcal{L}_\epsilon^{\widehat{u}_k,u_j,\overline{\phi}_k^{\widehat{u}_k},\phi_j}\psi_k(t,x_k).
\end{aligned} \tag{A.14}$$

Then the definition of infinitesimal generator in Eq (9) can be interpreted as

$$\begin{aligned}
\mathcal{L}^{\widehat{u}_k,u_j,\underline{\phi}_k^{\widehat{u}_k},\phi_j}\psi_k(t,x_k) &= \lim_{\epsilon\downarrow 0} \frac{\mathcal{L}_\epsilon^{\widehat{u}_k,u_j,\underline{\phi}_k^{u_k^\epsilon},\phi_j}\psi_k(t,x_k)}{\epsilon}, \\
\mathcal{L}^{\widehat{u}_k,u_j,\overline{\phi}_k^{\widehat{u}_k},\phi_j}\psi_k(t,x_k) &= \lim_{\epsilon\downarrow 0} \frac{\mathcal{L}_\epsilon^{\widehat{u}_k,u_j,\overline{\phi}_k^{u_k^\epsilon},\phi_j}\psi_k(t,x_k)}{\epsilon}
\end{aligned} \tag{A.15}$$

Plugging Eq (A.14) into Eq (A.13), N_ϵ becomes

$$
\begin{aligned}
N_\epsilon =&\alpha \mathcal{L}_\epsilon^{\widehat{u}_k, u_j, \underline{\phi}_k^{\widehat{u}_k}, \phi_j} J_k^{u_k^*, u_j^*, \underline{\phi}_k^*, \underline{\phi}_j^*}(t, x_k) - \frac{\alpha\gamma_k}{2} \mathcal{L}_\epsilon^{\widehat{u}_k, u_j, \underline{\phi}_k^{\widehat{u}_k}, \phi_j} \underline{g}_k^2(t, x_k) \\
&+ \frac{\alpha\gamma_k}{2} \left\{ \left(\mathbb{E}_{t,x_k}^{\underline{\phi}_k^{\hat{u}_k}, \underline{\phi}_j^*} \left[\underline{g}_k\big(t+\epsilon, X_k^{\hat{u}_k}(t+\epsilon)\big) \right] \right)^2 - \underline{g}_k^2(t, x_k) \right\} \\
&+ \widehat{\alpha} \mathcal{L}_\epsilon^{\widehat{u}_k, u_j, \overline{\phi}_k^{\widehat{u}_k}, \phi_j} J_k^{u_k^*, u_j^*, \overline{\phi}_k^*, \overline{\phi}_j^*}(t, x_k) - \frac{\widehat{\alpha}\gamma_k}{2} \mathcal{L}_\epsilon^{\widehat{u}_k, u_j, \overline{\phi}_k^{\widehat{u}_k}, \phi_j} \overline{g}_k^2(t, x_k) \\
&+ \frac{\widehat{\alpha}\gamma_k}{2} \left\{ \left(\mathbb{E}_{t,x_k}^{\overline{\phi}_k^{\hat{u}_k}, \overline{\phi}_j^*} \left[\overline{g}_k\big(t+\epsilon, X_k^{\hat{u}_k}(t+\epsilon)\big) \right] \right)^2 - \overline{g}_k^2(t, x_k) \right\} \\
&+ \alpha \int_t^{t+\epsilon} h_\beta(\underline{\phi}_k^{\hat{u}_k}(s))\, ds - \widehat{\alpha} \int_t^{t+\epsilon} h_\beta(\overline{\phi}_k^{\hat{u}_k}(s))\, ds.
\end{aligned} \tag{A.16}
$$

According to Dynkin's formula, we have

$$
\mathbb{E}_{t,x_k}^{\underline{\phi}_k^{\hat{u}_k}, \underline{\phi}_j^*} \left[\underline{g}_k\big(t+\epsilon, X_k^{\hat{u}_k}(t+\epsilon)\big) \right] = g_k(t, x_k) + \mathbb{E}_{t,x_k}^{\underline{\phi}_k^{\hat{u}_k}, \underline{\phi}_j^*} \left[\int_t^{t+\epsilon} \mathcal{L}^{\widehat{u}_k, u_j^*, \underline{\phi}_k^{\widehat{u}_k}, \underline{\phi}_j^*} \underline{g}_k(s, X_k^{\widehat{u}_k, u_j^*}(s)) ds \right],
$$

and this indicates that

$$
\begin{aligned}
&\left(\mathbb{E}_{t,x_k}^{\underline{\phi}_k^{\hat{u}_k}, \underline{\phi}_j^*} \left[\underline{g}_k\big(t+\epsilon, X_k^{\hat{u}_k}(t+\epsilon)\big) \right] \right)^2 - \underline{g}_k^2(t, x_k) \\
=&2\underline{g}_k(t, x_k) \mathbb{E}_{t,x_k}^{\underline{\phi}_k^{\hat{u}_k}, \underline{\phi}_j^*} \left[\int_t^{t+\epsilon} \mathcal{L}^{\widehat{u}_k, u_j^*, \underline{\phi}_k^{\widehat{u}_k}, \underline{\phi}_j^*} \underline{g}_k(s, X_k^{\widehat{u}_k, u_j^*}(s)) ds \right] + o(\epsilon).
\end{aligned} \tag{A.17}
$$

Similarly, we have

$$
\begin{aligned}
&\left(\mathbb{E}_{t,x_k}^{\overline{\phi}_k^{\hat{u}_k}, \overline{\phi}_j^*} \left[\overline{g}_k\big(t+\epsilon, X_k^{\hat{u}_k}(t+\epsilon)\big) \right] \right)^2 - \overline{g}_k^2(t, x_k) \\
=&2\overline{g}_k(t, x_k) \mathbb{E}_{t,x_k}^{\overline{\phi}_k^{\hat{u}_k}, \overline{\phi}_j^*} \left[\int_t^{t+\epsilon} \mathcal{L}^{\widehat{u}_k, u_j^*, \overline{\phi}_k^{\widehat{u}_k}, \overline{\phi}_j^*} \overline{g}_k(s, X_k^{\widehat{u}_k, u_j^*}(s)) ds \right] + o(\epsilon).
\end{aligned} \tag{A.18}
$$

Plugging Eq (A.17) and Eq (A.18) into Eq (A.16), we obtain

$$
\begin{aligned}
N_\epsilon =&\alpha \mathcal{L}_\epsilon^{\widehat{u}_k, u_j, \underline{\phi}_k^{\widehat{u}_k}, \phi_j} J_k^{u_k^*, u_j^*, \underline{\phi}_k^*, \underline{\phi}_j^*}(t, x_k) - \frac{\alpha\gamma_k}{2} \mathcal{L}_\epsilon^{\widehat{u}_k, u_j, \underline{\phi}_k^{\widehat{u}_k}, \phi_j} \underline{g}_k^2(t, x_k) \\
&+ \alpha\gamma_k \underline{g}_k(t, x_k) \mathbb{E}_{t,x_k}^{\underline{\phi}_k^{\hat{u}_k}, \underline{\phi}_j^*} \left[\int_t^{t+\epsilon} \mathcal{L}^{\widehat{u}_k, u_j^*, \underline{\phi}_k^{\widehat{u}_k}, \underline{\phi}_j^*} \underline{g}_k(s, X_k^{\widehat{u}_k, u_j^*}(s)) ds \right] \\
&+ \widehat{\alpha} \mathcal{L}_\epsilon^{\widehat{u}_k, u_j, \overline{\phi}_k^{\widehat{u}_k}, \phi_j} J_k^{u_k^*, u_j^*, \overline{\phi}_k^*, \overline{\phi}_j^*}(t, x_k) - \frac{\widehat{\alpha}\gamma_k}{2} \mathcal{L}_\epsilon^{\widehat{u}_k, u_j, \overline{\phi}_k^{\widehat{u}_k}, \phi_j} \overline{g}_k^2(t, x_k) \\
&+ \widehat{\alpha}\gamma_k \overline{g}_k(t, x_k) \mathbb{E}_{t,x_k}^{\overline{\phi}_k^{\hat{u}_k}, \overline{\phi}_j^*} \left[\int_t^{t+\epsilon} \mathcal{L}^{\widehat{u}_k, u_j^*, \overline{\phi}_k^{\widehat{u}_k}, \overline{\phi}_j^*} \overline{g}_k(s, X_k^{\widehat{u}_k, u_j^*}(s)) ds \right] \\
&+ \alpha \int_t^{t+\epsilon} h_\beta(\underline{\phi}_k^{\hat{u}_k}(s))\, ds - \widehat{\alpha} \int_t^{t+\epsilon} h_\beta(\overline{\phi}_k^{\hat{u}_k}(s))\, ds + o(\epsilon).
\end{aligned} \tag{A.19}
$$

Eq (A.7) implies that

$$
\begin{aligned}
&\alpha_k \mathcal{L}^{\widehat{u}_k, u_j^*, \underline{\phi}_k^{\widehat{u}_k}, \underline{\phi}_j^*} V_k(t, x_k) + \widehat{\alpha}_k \mathcal{L}^{\widehat{u}_k, u_j^*, \overline{\phi}_k^{\widehat{u}_k}, \overline{\phi}_j^*} V_k(t, x_k) \\
=&\alpha_k \mathcal{L}^{\widehat{u}_k, u_j^*, \underline{\phi}_k^{\widehat{u}_k}, \underline{\phi}_j^*} \left[\alpha_k \underline{J}_k^{u_k^*, \underline{\phi}_k^*, u_j^*, \underline{\phi}_j^*}(t, x_k) + \widehat{\alpha}_k \overline{J}_k^{u_k^*, \overline{\phi}_k^*, u_j^*, \overline{\phi}_j^*}(t, x_k) \right] \\
&+ \widehat{\alpha}_k \mathcal{L}^{\widehat{u}_k, u_j^*, \overline{\phi}_k^{\widehat{u}_k}, \overline{\phi}_j^*} \left[\alpha_k \underline{J}_k^{u_k^*, \underline{\phi}_k^*, u_j^*, \underline{\phi}_j^*}(t, x_k) + \widehat{\alpha}_k \overline{J}_k^{u_k^*, \overline{\phi}_k^*, u_j^*, \overline{\phi}_j^*}(t, x_k) \right] \\
=&\alpha_k \widehat{\alpha}_k (\mathcal{L}^{\widehat{u}_k, u_j^*, \underline{\phi}_k^{\widehat{u}_k}, \underline{\phi}_j^*} - \mathcal{L}^{\widehat{u}_k, u_j^*, \overline{\phi}_k^{\widehat{u}_k}, \overline{\phi}_j^*}) \left[\overline{J}_k^{u_k^*, \overline{\phi}_k^*, u_j^*, \overline{\phi}_j^*}(t, x_k) - \underline{J}_k^{u_k^*, \underline{\phi}_k^*, u_j^*, \underline{\phi}_j^*}(t, x_k) \right] \\
&+ \alpha_k \mathcal{L}^{\widehat{u}_k, u_j^*, \underline{\phi}_k^{\widehat{u}_k}, \underline{\phi}_j^*} \underline{J}_k^{u_k^*, \underline{\phi}_k^*, u_j^*, \underline{\phi}_j^*}(t, x_k) + \widehat{\alpha}_k \mathcal{L}^{\widehat{u}_k, u_j^*, \overline{\phi}_k^{\widehat{u}_k}, \overline{\phi}_j^*} \overline{J}_k^{u_k^*, \overline{\phi}_k^*, u_j^*, \overline{\phi}_j^*}(t, x_k).
\end{aligned}
$$

As $\overline{J}_k^{u_k^*, \overline{\phi}_k^*, u_j^*, \overline{\phi}_j^*}(t, x_k) - \underline{J}_k^{u_k^*, \underline{\phi}_k^*, u_j^*, \underline{\phi}_j^*}(t, x_k)$ is independent of x_k, we have

$$
\begin{aligned}
&\alpha_k \mathcal{L}^{\widehat{u}_k, u_j^*, \underline{\phi}_k^{\widehat{u}_k}, \underline{\phi}_j^*} V_k(t, x_k) + \widehat{\alpha}_k \mathcal{L}^{\widehat{u}_k, u_j^*, \overline{\phi}_k^{\widehat{u}_k}, \overline{\phi}_j^*} V_k(t, x_k) \\
=&\alpha_k \mathcal{L}^{\widehat{u}_k, u_j^*, \underline{\phi}_k^{\widehat{u}_k}, \underline{\phi}_j^*} \underline{J}_k^{u_k^*, \underline{\phi}_k^*, u_j^*, \underline{\phi}_j^*}(t, x_k) + \widehat{\alpha}_k \mathcal{L}^{\widehat{u}_k, u_j^*, \overline{\phi}_k^{\widehat{u}_k}, \overline{\phi}_j^*} \overline{J}_k^{u_k^*, \overline{\phi}_k^*, u_j^*, \overline{\phi}_j^*}(t, x_k).
\end{aligned}
\tag{A.20}
$$

From the extended HJB equation in Eq (9), we have

$$
\begin{aligned}
\alpha_k \Big[&\mathcal{L}^{\widehat{u}_k, u_j^*, \underline{\phi}_k^{\widehat{u}_k}, \underline{\phi}_j^*} V_k(t, x_k) - \frac{\gamma_k}{2} \mathcal{L}^{\widehat{u}_k, u_j^*, \underline{\phi}_k^{\widehat{u}_k}, \underline{\phi}_j^*} \underline{g}_k^2(t, x_k) \\
&+ \gamma_k \underline{g}_k(t, x_k) \mathcal{L}^{\widehat{u}_k, u_j^*, \underline{\phi}_k^{\widehat{u}_k}, \underline{\phi}_j^*} \underline{g}_k(t, x_k) + h_\beta(\underline{\phi}_k^{\hat{u}_k}(t)) \Big] \\
+\widehat{\alpha}_k \Big[&\mathcal{L}^{\widehat{u}_k, u_j^*, \overline{\phi}_k^{\widehat{u}_k}, \overline{\phi}_j^*} V_k(t, x_k) - \frac{\gamma_k}{2} \mathcal{L}^{\widehat{u}_k, u_j^*, \overline{\phi}_k^{\widehat{u}_k}, \overline{\phi}_j^*} \overline{g}_k^2(t, x_k) \\
&+ \gamma_k \overline{g}_k(t, x_k) \mathcal{L}^{\widehat{u}_k, u_j^*, \overline{\phi}_k^{\widehat{u}_k}, \overline{\phi}_j^* *} \overline{g}_k(t, x_k) - h_\beta(\overline{\phi}_k^{\hat{u}_k}(t)) \Big] \leq 0.
\end{aligned}
$$

Substituting Eq (A.20) into the last inequality yields

$$
\begin{aligned}
\alpha_k \Big[&\mathcal{L}^{\widehat{u}_k, u_j^*, \underline{\phi}_k^{\widehat{u}_k}, \underline{\phi}_j^*} J_k^{u_k^*, \underline{\phi}_k^*, u_j^*, \underline{\phi}_j^*}(t, x_k) - \frac{\gamma_k}{2} \mathcal{L}^{\widehat{u}_k, u_j^*, \underline{\phi}_k^{\widehat{u}_k}, \underline{\phi}_j^*} \underline{g}_k^2(t, x_k) \\
&+ \gamma_k \underline{g}_k(t, x_k) \mathcal{L}^{\widehat{u}_k, u_j^*, \underline{\phi}_k^{\widehat{u}_k}, \underline{\phi}_j^*} \underline{g}_k(t, x_k) + h_\beta(\underline{\phi}_k^{\hat{u}_k}(t)) \Big] \\
+\widehat{\alpha}_k \Big[&\mathcal{L}^{\widehat{u}_k, u_j^*, \overline{\phi}_k^{\widehat{u}_k}, \underline{\phi}_j^*} J_k^{u_k^*, \overline{\phi}_k^*, u_j^*, \overline{\phi}_j^*}(t, x_k) - \frac{\gamma_k}{2} \mathcal{L}^{\widehat{u}_k, u_j^*, \overline{\phi}_k^{\widehat{u}_k}, \overline{\phi}_j^*} \overline{g}_k^2(t, x_k) \\
&+ \gamma_k \underline{g}_k(t, x_k) \mathcal{L}^{\widehat{u}_k, u_j^*, \overline{\phi}_k^{\widehat{u}_k}, \overline{\phi}_j^* *} \overline{g}_k(t, x_k) - h_\beta(\overline{\phi}_k^{\hat{u}_k}(t)) \Big] \leq 0.
\end{aligned}
$$

Using Eq (A.15), we have that $N_\epsilon \leq o(\epsilon)$. Therefore, u_k^* is a reinsurance-investment equilibrium strategy. □

References

1. Li, B., Li, D., and Xiong, D. (2016). Alpha-robust mean-variance reinsurance-investment strategy. Journal of Economic Dynamics and Control, 70, 101-123.
