# Supplementary material for: Reinsurance–investment game between two α-maxmin mean–variance insurers
Source: PLoS One. 2025 Jun 27;20(6):e0326125. doi: 10.1371/journal.pone.0326125 (PMC12204578; doi:10.1371/journal.pone.0326125)
Supplement: S1 Table — (PDF) [file pone.0326125.s002.pdf]

Minimal data set

Values of basic parameters.

common	t	T	r	μ	σ	ρ			
	0	10	0.05	0.1	0.6	0.5			
insurer 1	n_1	θ_1	η_1	γ_1	μ_1	σ_1	α_1	β_{11}	β_{12}
	0.4	0.15	0.2	0.5	7	9	0.6	0.4	0.6
insurer 2	n_2	θ_2	η_2	γ_2	μ_2	σ_2	α_2	β_{21}	β_{22}
	0.8	0.2	0.3	0.9	12	8	0.8	0.5	0.7
