# Supplementary figures and images for: Reinsurance–investment game between two α-maxmin mean–variance insurers
Source: PLoS One. 2025 Jun 27;20(6):e0326125. doi: 10.1371/journal.pone.0326125 (PMC12204578; doi:10.1371/journal.pone.0326125)

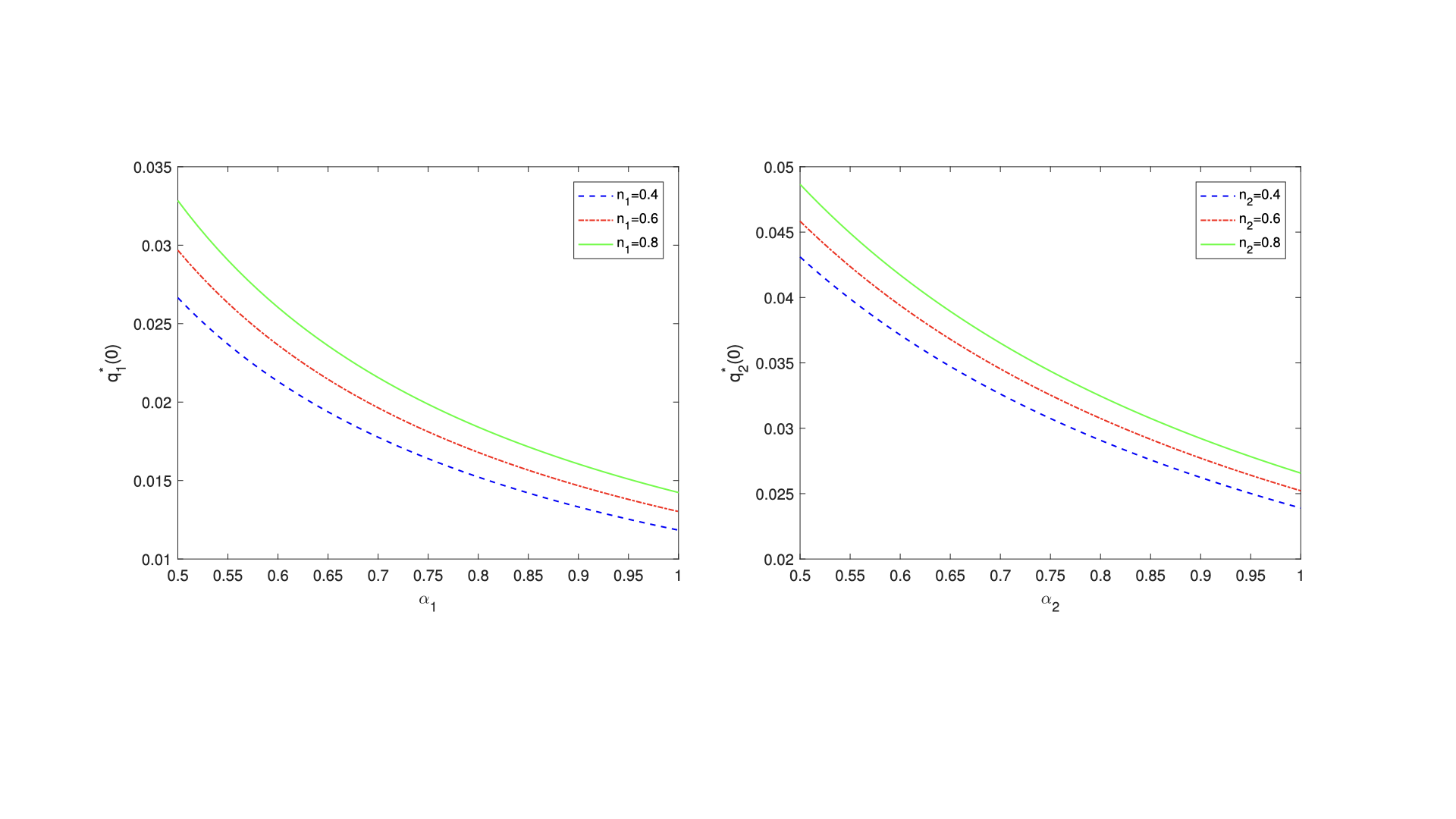

Supplement: S1 Fig — (TIF) [file pone.0326125.s003.tiff]

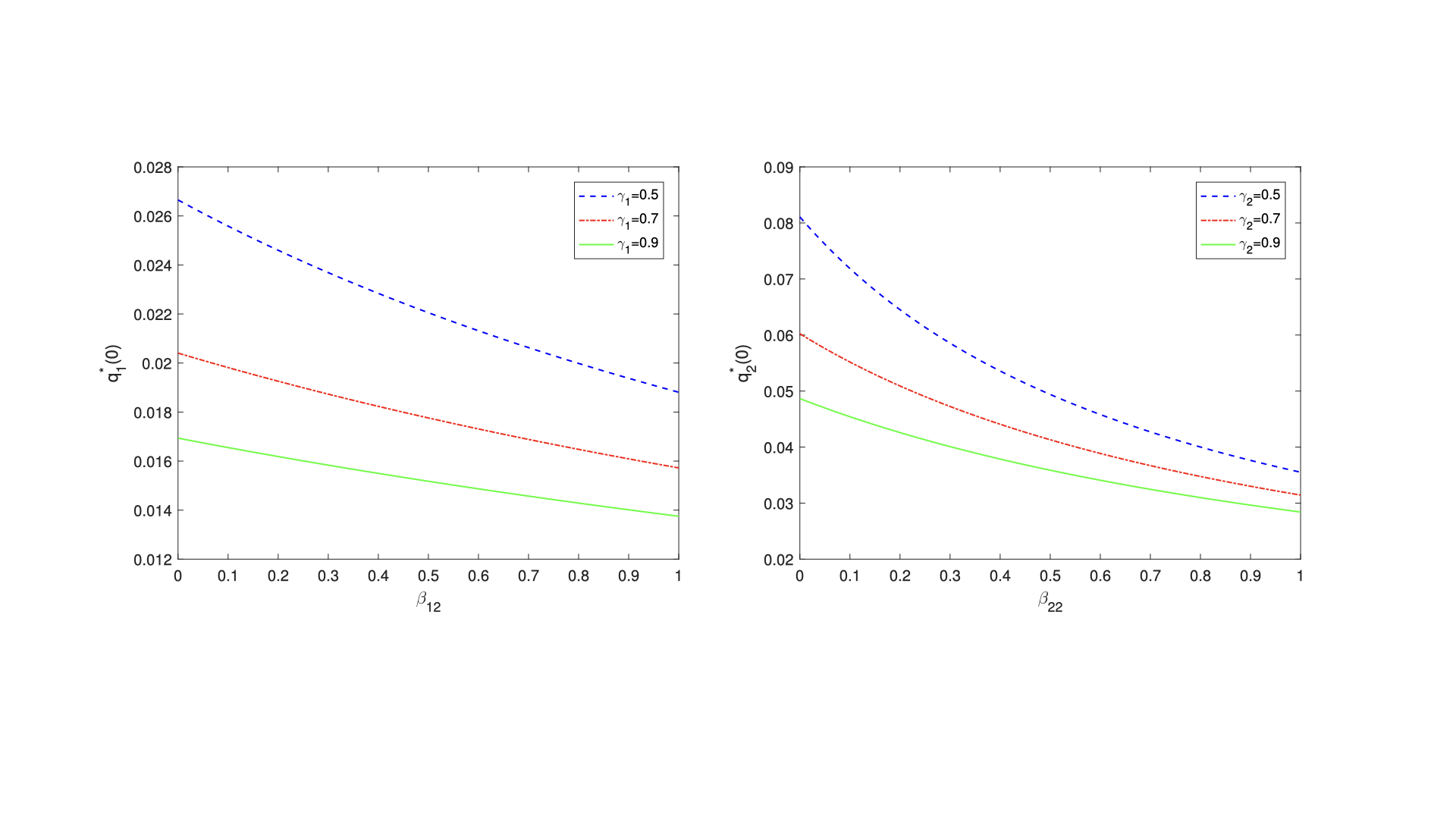

Supplement: S2 Fig — (TIF) [file pone.0326125.s004.tiff]

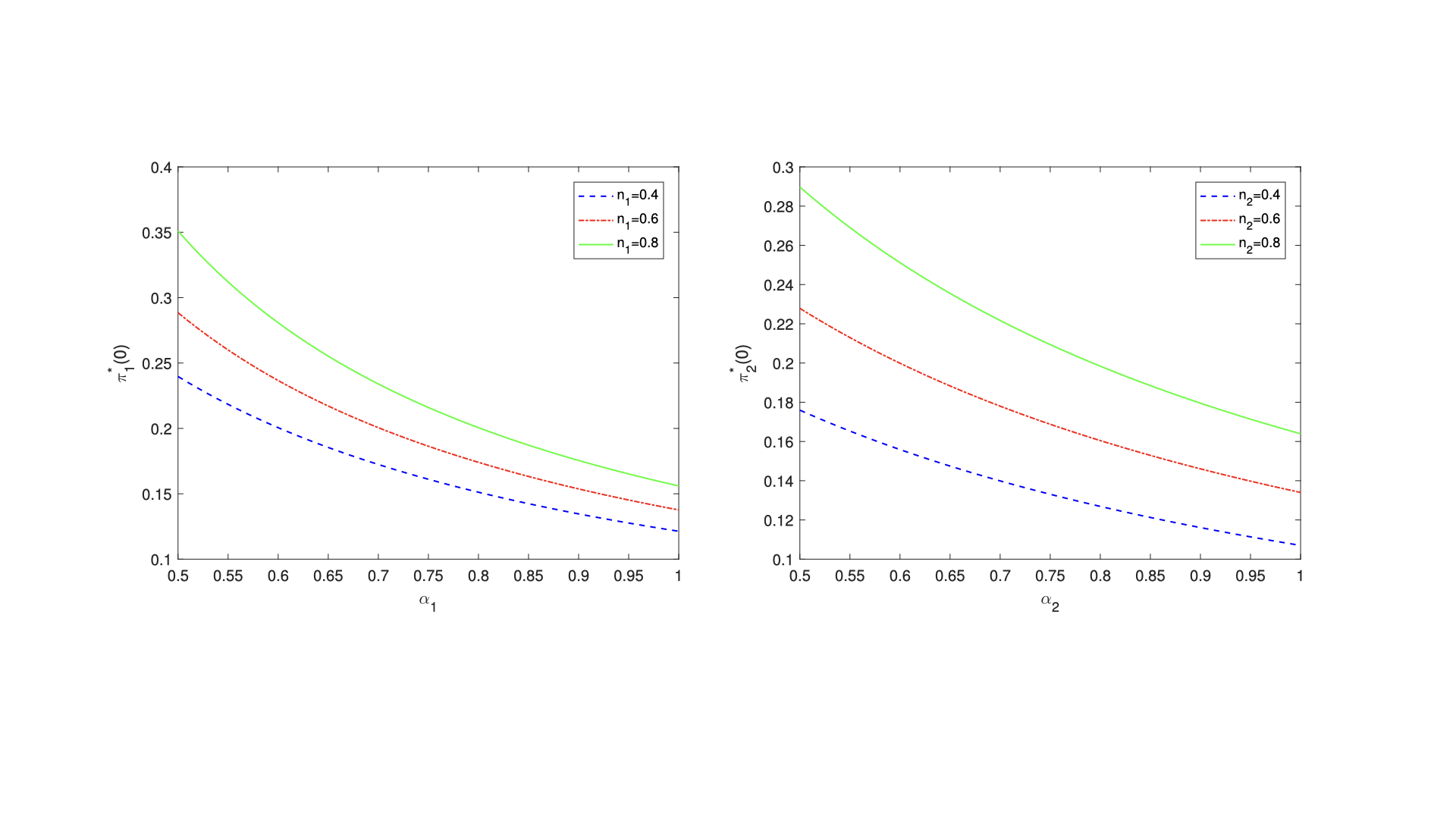

Supplement: S3 Fig — (TIF) [file pone.0326125.s005.tiff]

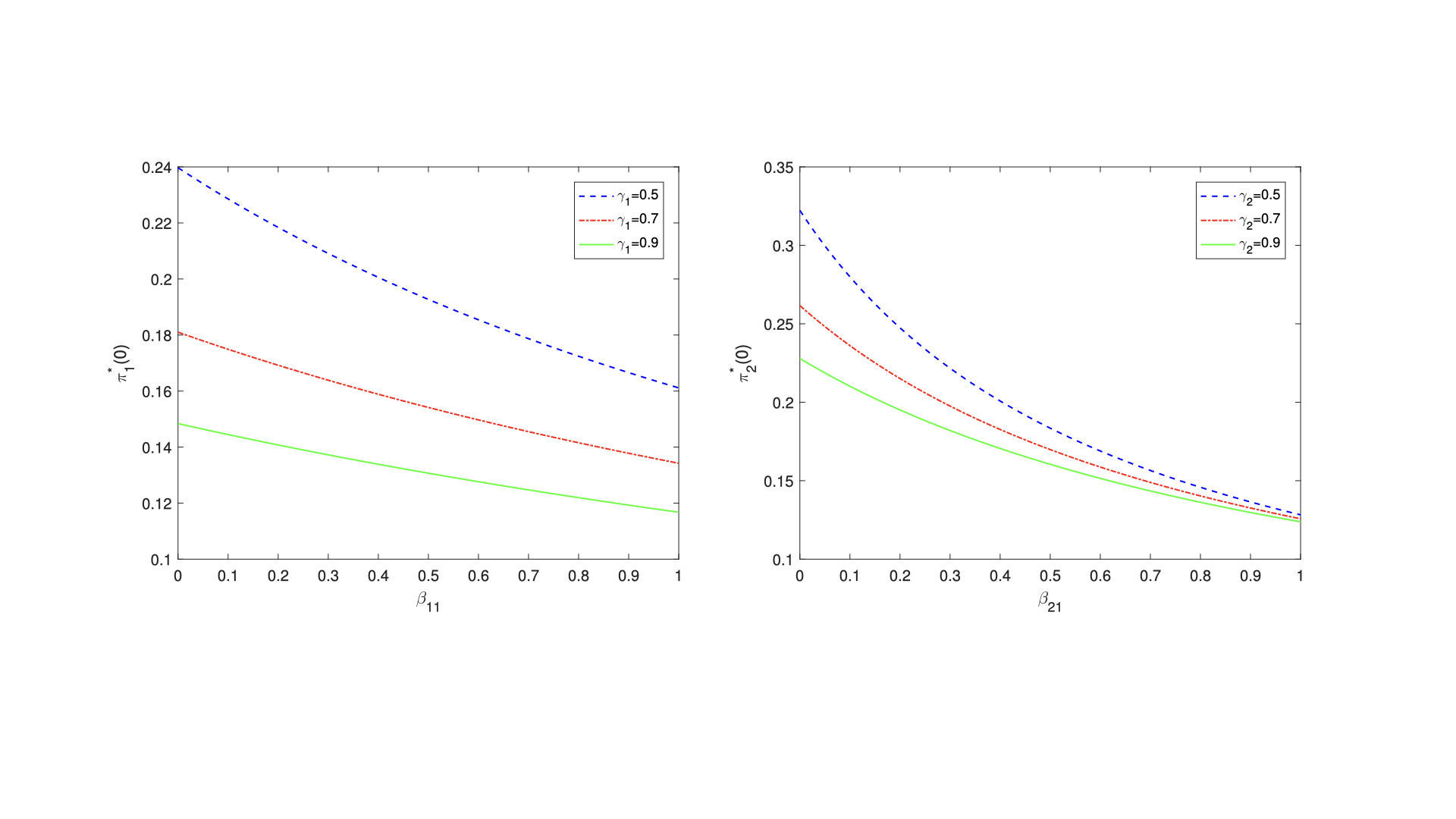

Supplement: S4 Fig — (TIF) [file pone.0326125.s006.tiff]
